# scDFN: enhancing single-cell RNA-seq clustering with deep fusion networks

**DOI:** 10.1093/bib/bbae486

**Published:** 2024-10-05

**Authors:** Tianxiang Liu, Cangzhi Jia, Yue Bi, Xudong Guo, Quan Zou, Fuyi Li

**Affiliations:** School of Science, Dalian Maritime University, 1 Linghai Road, Dalian 116026, China; School of Science, Dalian Maritime University, 1 Linghai Road, Dalian 116026, China; Department of Biochemistry and Molecular Biology, Biomedicine Discovery Institute, Monash University, Melbourne, VIC 3800, Australia; College of Information Engineering, Northwest A&F University, No. 3 Taicheng Road, Yangling, Shaanxi,China; Institute of Fundamental and Frontier Sciences, University of Electronic Science and Technology of China, No. 2006, Xiyuan Ave, West Hi-Tech Zone, 611731, Chengdu, Sichuan, China; College of Information Engineering, Northwest A&F University, No. 3 Taicheng Road, Yangling, Shaanxi,China; South Australian Immunogenomics Cancer Institute, The University of Adelaide, 4 North Terrace, SA 5000, Australia

**Keywords:** single-cell RNA sequencing, clustering, deep learning, autoencoder

## Abstract

Single-cell ribonucleic acid sequencing (scRNA-seq) technology can be used to perform high-resolution analysis of the transcriptomes of individual cells. Therefore, its application has gained popularity for accurately analyzing the ever-increasing content of heterogeneous single-cell datasets. Central to interpreting scRNA-seq data is the clustering of cells to decipher transcriptomic diversity and infer cell behavior patterns. However, its complexity necessitates the application of advanced methodologies capable of resolving the inherent heterogeneity and limited gene expression characteristics of single-cell data. Herein, we introduce a novel deep learning-based algorithm for single-cell clustering, designated scDFN, which can significantly enhance the clustering of scRNA-seq data through a fusion network strategy. The scDFN algorithm applies a dual mechanism involving an autoencoder to extract attribute information and an improved graph autoencoder to capture topological nuances, integrated via a cross-network information fusion mechanism complemented by a triple self-supervision strategy. This fusion is optimized through a holistic consideration of four distinct loss functions. A comparative analysis with five leading scRNA-seq clustering methodologies across multiple datasets revealed the superiority of scDFN, as determined by better the Normalized Mutual Information (NMI) and the Adjusted Rand Index (ARI) metrics. Additionally, scDFN demonstrated robust multi-cluster dataset performance and exceptional resilience to batch effects. Ablation studies highlighted the key roles of the autoencoder and the improved graph autoencoder components, along with the critical contribution of the four joint loss functions to the overall efficacy of the algorithm. Through these advancements, scDFN set a new benchmark in single-cell clustering and can be used as an effective tool for the nuanced analysis of single-cell transcriptomics.

## Introduction

Single-cell ribonucleic acid sequencing (scRNA-seq) technology represents a considerable advancement in high-throughput technology. It can be used to analyze the transcriptome of individual cells at the finest level [1]. This technique differs from conventional bulk RNA-seq methodologies, which miss the intricate heterogeneity among cells [2]. The ever-increasing content of heterogeneous single-cell data highlights the need for the development of efficient tools specifically for conducting single-cell transcriptome analysis [3]. Cell clustering is an important technique that can be used to determine cell heterogeneity and perform cell development trajectory analysis [4], as well as the KEGG pathway analysis and gene ontology (GO) analysis. By grouping cells based on their expression matrices, clustering facilitates the elucidation of internal structural information and molecular profiles, thus, influencing the quality of downstream single-cell transcriptomics analyses. Single-cell clustering, however, is hindered by the high heterogeneity and low gene expression rates of single-cell transcriptomics data.

Conventional clustering methods, such as *k*-means clustering [5], hierarchical clustering [6], principal component analysis (PCA), and K-nearest neighbors (KNNs), have been used to develop cell clustering approaches. For example, SC3 [7] uses genetic filtering along with PCA and Laplace transforms, enhancing *k*-means with hierarchical clustering to refine the clustering outcome by integrating the initial value and condition variations. This approach addresses the limitations of greedy algorithms by ensuring consistency. Additionally, approaches like phenotyping by accelerated refined community (PARC) [8] and Seurat [9] utilize KNN to allocate cells to their nearest clusters based on computational distances and neighbor counts, which can vary significantly. Tools for single cell analysis (TSCAN) is an unsupervised approach that links gene expression to the temporal or spatial orientations of cells, enabling cell clustering via trajectory inference analysis [10]. However, when dealing with large datasets, conventional clustering techniques struggle with scalability, and the efficacy of methods like KNN depends heavily on the choice of the definition of computational distance and the number of nearest neighbors.

Deep learning has introduced various sophisticated methods for clustering and analyzing scRNA-seq data. These advanced single-cell data clustering approaches are categorized into five main groups based on their network optimization objectives. These groups include generative adversarial network-based methods (e.g. scGPCL [11] and scDECL [12]), subspace clustering-based methods (e.g. scBGEDA [13]), Gaussian mixture model-based methods (e.g. scSSA [14]), spectral clustering-based methods (e.g. Secuer [15] and scDSSC [16]), and self-optimization-based methods (e.g. scziDesk [17], scDeepCluster [18], DESC [19], and GraphSCC [20]). The methods are described in Table 1. A summary of deep learning-based single-cell clustering methods. For example, scDSSC [16] applies an autoencoder for noise reduction and dimensionality reduction and performs spectral clustering through a sparse self-expressive matrix. However, the model uses different hyperparameters for different datasets while training. DESC [19] obtains parameters through an automatic encoder and learns the spatial structure by iteratively optimizing the clustering objective function. Additionally, DESC uses the traditional mean square error (MSE) loss as the data reconstruction error and ignores the distance between similar cells; thus, it cannot maintain the global and local structure of the data. DCA [21] uses a deep count autoencoder network to denoise the scRNA-seq data. In scDeepCluster [18], autoencoders are trained to denoise the data by simultaneously reducing reconstruction loss and separation loss, which helps in learning transcriptome expression and clustering features simultaneously. However, as it does not pre-select highly variable genes as input features, it has low clustering accuracy, is time-consuming, and requires a lot of memory. The scSSA [14] uses a semi-supervised autoencoder and fastICA for dimensionality reduction and constructs a Gaussian mixture model that yields accurate clustering results. However, different epochs may be required to minimize the loss in different datasets. Because it uses a semi-supervised autoencoder, at least some cells need to be labeled while training. Otherwise, the clustering performance may be greatly affected. The scziDesk [17] model combines deep learning techniques with denoising autoencoders to characterize scRNA-seq data, and a soft self-training *k*-means algorithm is used to cluster cell populations in the potential learning space. The scBGEDA [13] performs single-cell clustering by a dual denoising autoencoder and bipartite graph ensemble clustering algorithm. Most of the models mentioned above use *k*-means clustering for initialization, and the results are optimized based on clustering loss. However, they ignore the structural information among cells and face difficulties while dealing with large datasets. In scTAG [22], a topologically adaptive graph convolutional autoencoder is developed to learn cell–cell topological representations. A hypothesis-free cell learning framework, known as scGNN [23], uses graph neural networks to characterize the relationship between cells. To preserve the topological structure and attribute information of the scRNA-seq data, multi-task-oriented graph autoencoders are applied by scGAE [24] for dimensionality reduction. GraphSCC [20] combines graph convolutional networks (GCNs) and denoising autoencoders to integrate the structural information of scRNA-seq data, and a dual self-supervised module is designed to optimize the latent representation. In scMGCA [25], a graph-embedded autoencoder is constructed to learn cell–cell topological representations and cluster assignments simultaneously. Notably, Ding et al [26] provides an end-to-end toolkit DANCE, which supports 3 modules and 8 tasks with 32 state-of-art methods on 21 benchmark datasets. However, graph autoencoders often miss key patterns in gene expression data, which is a major limitation of these methods.

**Table 1:** A summary of deep learning-based single-cell clustering methods

Method classification	Tool	Year	Model	Objective	Autoencoder	Hyperparameters consistency?
Generative adversarial network-based	scGPCL	2023	Graph prototypical contrastive learning	Clustering	GNN-based autoencoder	No
	scDECL	2023	Contrastive learning and pairwise constraints	Clustering	Deep autoencoder	No
Subspace clustering-based	scBGEDA	2023	Dual denoising autoencoder with bipartite graph ensemble clustering	Clustering and interpretable analysis	Denoising autoencoder	Yes
Gaussian mixture model-based	scSSA	2022	Semi-supervised autoencoder	Clustering	Deep autoencoder	Yes
Spectral clustering-based	Secuer	2022	Construct weighted bipartite graph, k-means clustering	Clustering	None	No
	scDSSC	2022	Autoencoder, subspace clustering	Clustering and interpretable analysis	Denoising autoencoder	No
Self-optimization–based methods	scDeepCluster	2019	Autoencoder	Clustering	Denoising autoencoder	No
	DCA	2019	Deep count autoencoder	Clustering and interpretable analysis	Deep autoencoder	No
	scziDesk	2020	Autoencoder, soft K-means clustering	Clustering and interpretable analysis	Deep autoencoder	No
	DESC	2020	Stacked autoencoder, iterative clustering	Clustering and interpretable analysis	Stacked autoencoder	Yes
	GraphSCC	2020	GCN, denoising autoencoder	Clustering and interpretable analysis	Denoising autoencoder	Yes
	scGNN	2021	Graph neural networks, left-truncated mixture Gaussian model	Clustering and interpretable analysis	Deep autoencoder and graph autoencoder	Yes
	scGAE	2021	Multitask-oriented graph autoencoder	Clustering and interpretable analysis	Graph autoencoder	Yes
	scTAG	2022	Embedding clustering	Clustering	Deep graph convolutional autoencoder	Yes
	scMGCA	2023	Graph-embedded autoencoder	Clustering and interpretable analysis	Graph autoencoder	Yes
	scDEFR	2023	Fusion cell topology encoder, transcriptomics profile-based graph encoder	Clustering	Deep autoencoder and graph autoencoder	Yes

Based on these approaches, several deep graph clustering methods have been developed, which combine autoencoder and graph encoder to extract the structural and attribute information of graphs. This combination has advantages over an autoencoder or a graph autoencoder used alone. SDCN [27] integrates multiple representations and structures of the data based on a dual self-supervised combination of autoencoder and GCN. In this process, the autoencoder and GCN help each other to improve clustering performance. DFCN [28] captures the shared representation of local and global information in input data by an autoencoder and a graph encoder. These results suggest that combining classic autoencoder and graph autoencoder is an effective method to extract cell information and cell–cell topology information of scRNA-seq data.

In this study, we propose a self-optimization deep single-cell clustering algorithm known as scDFN, which can extract the attribute information and structure information of scRNA-seq data through a fusion network. The scDFN has three main modules, including a data processing module, an information coding module, and an information fusion module. The data processing module is used to select highly variable genes and reduce the dimension of the features of the input scRNA-seq data. The information coding module extracts the cell representation information through the autoencoder and the improved graph autoencoder. This is the first study to apply the augmented graph network for the topological representation of data at the single-cell level. The information fusion module combines the extracted attribute and topology information based on the cross-network information fusion mechanism and the triple self-supervision strategy. Quadruple joint losses are adopted to optimize the cell clustering representation in the whole model. The results of a comprehensive evaluation and analysis confirmed that scDFN can improve scRNA-seq clustering effectively. Compared to five state-of-the-art scRNA-seq clustering methods, scDFN achieved better NMI [29], ARI [30], local inverse Simpson’s index (LISI), and average silhouette width (ASW) [31] on multiple datasets. Additionally, scDFN can be used to analyze multi-cluster datasets effectively. Ablation studies have not only shown the necessity of an autoencoder and an improved graph autoencoder but also revealed the importance of four joint losses. Our scDFN method also exhibits excellent robustness during clustering and effectively eliminates batch effects.

## Materials and methods

### Overall framework

**Figure 1:**
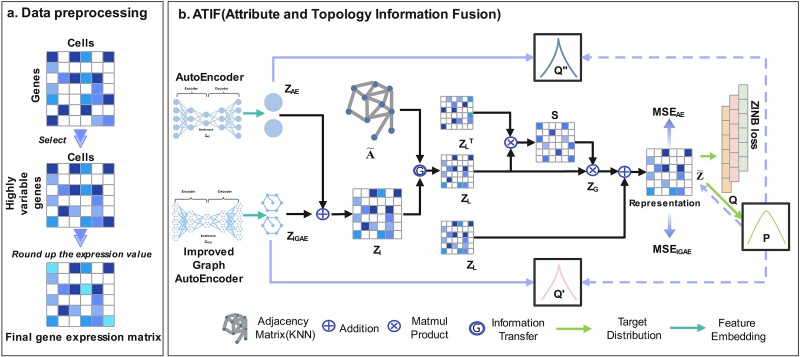
Network architecture of scDFN. (a) The data preprocessing module. (b) ATIF.

The scDFN method we developed mainly included three parts for optimizing clustering results by learning a low-dimensional representation of cells, whose overall framework is illustrated in Fig. 1. The first part involved data processing using the Python Scanpy package [32]. We selected ($M$, a highly variable gene, and plotted a KNN graph as the input of the improved graph autoencoder (IGAE). The second part involved the establishment of the framework of AE and IGAE to obtain a low-dimensional potential representation of cells. In this part, AE was used to integrate the attribute information of cells and obtain low-dimensional potential embedding. IGAE can be used to extract the main cell information and the topological structure of the cells by learning the expression matrix and the cell graph. The third part is the most important part that employed a zero-inflated negative binomial (ZINB) distribution not only to simulate the distribution of cells but also to act as a decoder to reconstruct the gene representations. The MSE reconstruction loss was used to reconstruct the cell representations of AE and IGAE. The self-optimizing clustering task based on the Kullback–Leibler (KL) divergence was performed on the embedded representation, which we called the triple self-monitoring mechanism. By simultaneously correcting clustering loss, ZINB-based loss, cell graph, and expression matrix reconstruction loss, we captured the optimal attribute information and topology information of cells.

### Data collection and processing

We collected 32 real scRNA-seq datasets of mouse and human tissues. The cell types ranged from 2 to 49, and cell numbers ranged from 56 to 12 089 [33–52]. These datasets were obtained from four sequencing platforms, including inDrop, 10X, SMARTer, and Smart-seq2. These datasets are known labels for each cell and have been used to test performance in previous studies. Detailed information on the collected scRNA-seq datasets is provided in Supplementary Information and also summarized in Supplementary Table S1 available online at http://bib.oxfordjournals.org/. The scDFN model uses the original gene expression matrix $X\in{R}^{N\times d}$ as input, in which *N* represents the number of cells and $d$ represents the number of genes. By standardizing and compressing the raw gene expression matrix *X* (see Supplementary Information), we obtained the gene expression matrix ${X}^{\prime}\in{R}^{N\times{d}^{\prime }},{d}^{\prime }<d$.

### Improved graph autoencoder

Given an undirected graph $G=\left\{V,E\right\}$, $V=\left\{{v}_1,{v}_2,\cdots, {v}_N\right\}$ represents the set of nodes, $E$ represents the set of edges, and $N$ represents the number of cells. The original adjacency matrix *A* of graph *G* is defined by:


(1) \begin{equation*} A={\left({a}_{ij}\right)}_{N\times N}=\Big\{{\displaystyle \begin{array}{@{}c}1,\\{}0,\end{array}}{\displaystyle \begin{array}{c} if\ \left({v}_i,{v}_i\right)\in E,\\{} else.\kern3em \end{array}} \end{equation*}

Let $D=\mathit{\operatorname{diag}}\left({d}_1,{d}_2,\dots, {d}_N\right)\in{R}^{N\times N}$ represents the degree matrix of *A*, and ${d}_i={\sum}_{v_j\in V}{a}_{ij}$.

Then, we used a novel symmetric IGAE to enhance stability by simultaneously reconstructing the attribute matrix and the adjacency matrix [28]. In IGAE, the encoder and decoder were modified as follows:


(2) \begin{equation*} {\displaystyle \begin{array}{l}{Z}^{(l)}=\sigma \left(\overline{A}{Z}^{\left(l-1\right)}{\hat{W}}^{(l)}\right)\\{}{\hat{Z}}^{(h)}=\sigma \left(\overline{A}{\hat{Z}}^{\left(h-1\right)}{W}^{(h)}\right)\end{array}} \end{equation*}

here, $\overline{A}={D}^{-\frac{1}{2}}\left(A+I\right){D}^{-\frac{1}{2}}$, $I\in{R}^{N\times N}$ represents an identity matrix, ${W}^{(l)}$ and ${W}^{(h)}$ represent the parameters that can be learned by the *l*-th coding layer and the *h*-th decoding layer, respectively, and $\sigma$ represents a linear activation function. The loss ${L}_w$ and ${L}_a$ can be expressed as follows:


(3) \begin{equation*} {L}_w=\frac{1}{2N}\left\Vert \overline{A}X-\hat{Z}\right\Vert \end{equation*}

(4) \begin{equation*} {L}_a=\frac{1}{2N}{\left\Vert \overline{A}-\hat{A}\right\Vert}_F^2 \end{equation*}

Here, $\hat{Z}$ represents the reconstructed weighted attribute matrix, and $\hat{A}$ represents the reconstructed *A* generated by an inner product operation with multi-level representations of the network [28]. For satisfactory cell representation, the mixed loss function was defined by the following formula:


(5) \begin{equation*} {L}_{IGAE}={L}_w+\gamma{L}_a \end{equation*}

where, $\gamma$ represents a hyperparameter that needs to be predefined to balance the weights of the two losses. In this study, we set $\gamma =0.1$. The goal of the network is to minimize the reconstruction loss of the weighted attribute matrix and the adjacency matrix simultaneously.

### Attribute and topology information fusion

The information fusion module was used to fully mine the feature attribute and topology information of the gene expression matrix, and an information fusion module of attribute information and topology information was proposed. The module consisted of two parts, including the cross-network information fusion mechanism and the triple self-supervision strategy. The overall structure of ATIF is shown in Fig. 1b.

First, the potential embedding ${Z}_{AE}\in{R}^{N\times{\mathrm{d}}^{\prime\prime}}$ obtained by the AE and the potential embedding ${Z}_{IGAE}\in{R}^{N\times{\mathrm{d}}^{\prime\prime}}$ obtained by the IGAE underwent initial information fusion; in the expressions, $d^{{\prime\prime} }$ represents the dimension of the potential embedded gene. The above-mentioned terms were combined as follows:


(6) \begin{equation*} {Z}_I=\alpha{Z}_{AE}+\left(1-\alpha \right){Z}_{IGAE} \end{equation*}

Here, $\alpha$ represents a learnable parameter. The value of $\alpha$ was initialized to 0.5, and then, automatically adjusted by the gradient descent method. Using a message-passing operation, we defined ${Z}_L=\overline{A}{Z}_I$, which was equivalent to a local data augmentation of the initial fused information.

Based on local data enhancement, we used an autocorrelation method to investigate the non-local relationship between cells through the autocorrelation matrix. Specifically, we first calculated the normalized autocorrelation matrix $S$, and the element ${S}_{ij}$ was defined as follows:


(7) \begin{equation*} {S}_{ij}=\frac{\exp \left({\left({Z}_L{Z}_L^T\right)}_{ij}\right)}{\sum_{k=1}^N\exp \left({\left({Z}_L{Z}_L^T\right)}_{ik}\right)}. \end{equation*}

Finally, we linked the global relationship and the local relationship matrices to ensure the smooth transmission of the initial fusion information


(8) \begin{equation*} \tilde{Z}=\beta \left(S{Z}_L\right)+{Z}_L. \end{equation*}

where $\beta$ is a learnable parameter.

The information fusion mechanism can be used to extract the global information and local information of cells from the aspects of local correlation and global correlation. The attribute information obtained from AE and the topological relationship of cells obtained from IGAE can achieve a better potential cell representation than that achieved by using either one alone.

### Triplet self-supervised strategy

We proposed a triple self-supervised module by integrating the AE and IGAE in the neural network architecture, the combination of which helped us extract the attribute information and topology information of the matrix. We trained these two modules to achieve end-to-end single-cell clustering. In the first step, to generate a more reliable clustering network for guiding training, we used a more stable clustering embedding $\tilde{Z}$ in Equation (8) to generate the target distribution. The *t*-distribution is used as the kernel to determine the similarity between the data representation ${\tilde{z}}_i$ and the cluster center vector ${u}_j$ [53]. The process can be expressed as follows:


(9) \begin{equation*} {q}_{ij}=\frac{{\left(1+\left\Vert{\tilde{z}}_i-{\mu}_j\right\Vert /v\right)}^{-\frac{v+1}{2}}}{\sum_{j^{\prime}}{\left(1+\left\Vert{\tilde{z}}_i-{\mu}_{j^{\prime}}\right\Vert /v\right)}^{-\frac{v+1}{2}}} \end{equation*}

Here, $v$ represents the degree of freedom of the Student’s distribution, ${q}_{ij}$ represents the probability of the $i$ -th node being assigned to the $j$ -th center (i.e. a soft assignment), and $Q=\left[{q}_{ij}\right]$ represents the distribution of all samples. In the second step, we optimized data representation by learning from high-confidence assignments. Specifically, we made the data representation closer to the cluster center, thereby improving the cohesion of the cluster. Thus, we introduced the target distribution $P$, which was calculated as follows:


(10) \begin{equation*} {p}_{ij}=\frac{q_{ij}^2/{\sum}_i{q}_{ij}}{\sum_{j^{\prime}}\left({q}_{i{j}^{\prime}}^2/{\sum}_i{q}_{i{j}^{\prime}}\right)} \end{equation*}

Here, $0\le{p}_{ij}\le 1$ represents the element that generates the target distribution $P\in{R}^{N\times K}$, which represents the probability of the $i$ -th sample belonging to the $j$ -th center. Each assignment in $Q$ was normalized by the sum of squares, so that the assignment had a higher confidence level, and the following objective function was obtained:


(11) \begin{equation*} {L}_{clu}= KL\left(P\big\Vert Q\right)=\sum \limits_i\sum \limits_j{p}_{ij}\log \frac{p_{ij}}{q_{ij}} \end{equation*}

Similar to the generated target distribution *Q*, we used Equation (10) to calculate the soft allocation distribution of AE and IGAE. On the potential embedding of the two sub-networks, we expressed the soft allocation distribution of IGAE and AE as ${Q}^{\prime}=\left[{q_{\mathrm{ij}}}^{\prime}\right]$ and ${Q}^{\prime\prime}=\left[{q_{ij}}^{\prime\prime}\right]$, respectively.

To train the network under a unified standard and improve the representation ability of each component, we designed a triple clustering loss using the following formula:


(12) \begin{equation*} {L}_{KL}={\sum}_i{\sum}_j{p}_{ij}\log \frac{p_{ij}}{\left({q}_{ij}+{q}_{ij}^{\prime }+{q}_{ij}^{{\prime\prime}}\right)/3} \end{equation*}

Through this loss, we aligned AE, IGAE, and their fusion distribution with the target distribution.

### Decoder based on zero-inflated negative binomial model

To capture the structure of the scRNA-seq data, we employed ZINB loss to the decoder. Specifically, ZINB is used to model the dropout event of scRNA-seq data based on zero components and the distribution of NB [21]. This method connects three independent connection layers with the last layer, and then estimates the parameters of ZINB, including the dropout rate $\pi$, dispersion degree $\theta$, and mean $\mu$. The output of the network is as follows:


(13) \begin{equation*} {\displaystyle \begin{array}{l}\varPi = sigmoid\left({W}_{\pi }X\right)\\{}M=\exp \left({W}_{\mu }X\right)\\{}\varTheta =\exp \left({W}_{\theta }X\right)\end{array}} \end{equation*}

Here, $W$ represents the learnable weight of the loss function and uses a ZINB-based decoder to reconstruct scRNA-seq data; NB and ZINB can be expressed as follows:


(14) \begin{equation*} NB\left(X|\mu, \theta \right)=\frac{\varGamma \left(X+\theta \right)}{X!\varGamma \left(\theta \right)}{\left(\frac{\theta }{\theta +\mu}\right)}^{\theta }{\left(\frac{\mu }{\theta +\mu}\right)}^x \end{equation*}

(15) \begin{equation*} ZINB\left(X|\pi, \mu, \theta \right)=\pi{\delta}_0(X)+\left(1-\pi \right) NB(X) \end{equation*}

The reconstruction loss function of the gene expression matrix $X$ is defined as the negative log-likelihood of the ZINB distribution and is expressed as follows:


(16) \begin{equation*} {L}_{ZINB}=-\log \left( ZINB\left(X|\pi, \mu, \sigma \right)\right) \end{equation*}

### Quadruple joint losses

The learning goal of the model has four main parts, including the reconstruction loss of AE (*L_AE_*), the double reconstruction loss of IGAE (*L_IGAE_*), the clustering loss of the triple self-monitoring mechanism (*L_KL_*), and the ZINB loss (*L_ZINB_*). These four losses are combined to optimize the clustering representation of cells jointly. The combined loss is expressed as follows:


(17) \begin{equation*} L={\gamma}_1{L}_{AE}+{\gamma}_2{L}_{IGAE}+{\gamma}_3{L}_{KL}+{\gamma}_4{L}_{ZINB} \end{equation*}

here, ${\gamma}_1,{\gamma}_2,{\gamma}_3$ represent predefined hyperparameters that balance clustering optimization and reconstruction losses, and ${\gamma}_4$ represents a hyperparameter that controls the data structure that captures the distribution of the scRNA-seq data. Various losses play certain roles in optimizing single-cell clustering representation (explained in the subsequent ablation analysis).

Details regarding model implementation and measurement indicators are provided in Supplementary Information.

## Results and discussion

### Performance comparisons with five state-of-the-art single-cell ribonucleic acid-sequencing clustering methods

**Figure 2:**
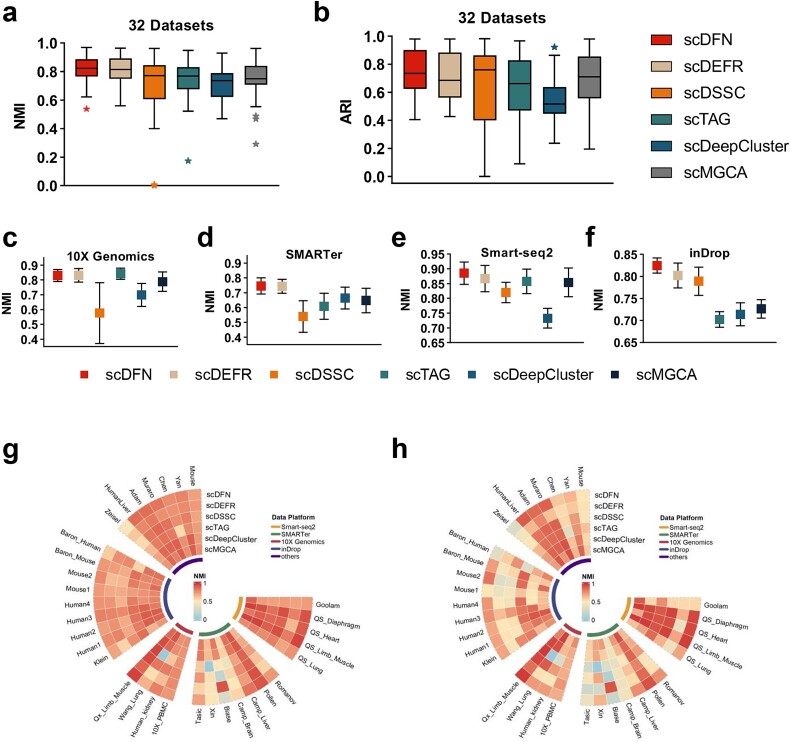
(a and b) A comparison of the average value of NMI and ARI on the 32 real scRNA-seq datasets among 6 clustering methods. (c–f) A comparison of the mean value of NMI on various data platforms among six clustering methods. (g and h) Specific numerical values for each method on each dataset.

We compared scDFN with five advanced single-cell clustering methods using 32 scRNA-seq datasets originating from multiple platforms to evaluate its clustering performance. These methods, including scMGCA, scDEFR, scTAG, scDeepCluster, and scDSSC, are the most advanced methods developed in recent years and have achieved good clustering performance. To ensure that the comparison was fair, the method of the baseline model was analyzed and found to be consistent with the original publication. The results of the comparison are summarized in Fig. 2 and Supplementary Tables S2 and S3 available online at http://bib.oxfordjournals.org/. We found that scDFN achieved the highest average scores of NMI (0.8131) and ARI (0.7518) on all 32 datasets, and scDFER achieved the second highest scores (NMI = 0.8161 and ARI = 0.7518; Fig. 2a and b). Specifically, the NMI score of scDFN performed well on 15 datasets, with the highest score of 0.9693 recorded for the QS_Diaphragm dataset [44], and scDFER performed well on seven datasets, with the best score of 0.9637 for the QS_Limb_Muscle dataset (Fig. 2g). The ARI value of scDFN performed well on 16 datasets, with the highest score of 0.9813 for the QS_Diaphragm dataset; the ARI value of the performance of scDSSC on six datasets was the second highest (Fig. 2h). We also compared the overall clustering performance (NMI and ARI) of the methods on various data platforms, including plate-based platform, flow cell-based platform, Smart-Seq 2, SMARTer, 10X Genomics, and inDrop (Fig. 2c–f). In addition, ASW and cLISI are also employed to evaluate the clustering performance (Figs S1 and S2; Supplementary Tables S4 and S5 available online at http://bib.oxfordjournals.org/). The cLISI scores of all clustering approaches are greater than 0.97, with the highest value of scDFN being 0.9967. For the ASW, scTAG is the best method, reaching 0.8769, while our scDFN only ranks fourth, reaching 0.5709. We also evaluated the computing time by scDFN and five other clustering algorithms on our PC. As shown in Table S6 and Fig. S4 (available online at http://bib.oxfordjournals.org/), the average running time of scDFN is 3.41 minutes on 32 datasets of varying sizes which is almost equivalent to that of scDSSC and scMGCA, more than that of scTAG and scDeepCluster, and much lower than that of scDEFR (Fig. S3 and Supplementary Table S6 available online at http://bib.oxfordjournals.org/). The experimental results showed that the clustering performance of the scMGCA algorithm on cross-platforms was better than the performance of the other five clustering algorithms, which confirmed the effectiveness and accuracy of the scMGCA algorithm in cross-platform clustering. It cannot be denied that our model performs poorly on certain datasets, such as Camp_Brain, Xin, and Romanov. The main reason may be that our model does not adjust parameters for each dataset and some datasets may contain a large amount of noise or irrelevant information. The clustering results on Camp_Brain and Romanov, all predicting methods perform not as well as others, which may be caused by the inherent nature of the data itself. The result analysis shows that the model needs further optimization in future work.

### Performance on multi-cluster datasets

**Figure 3:**
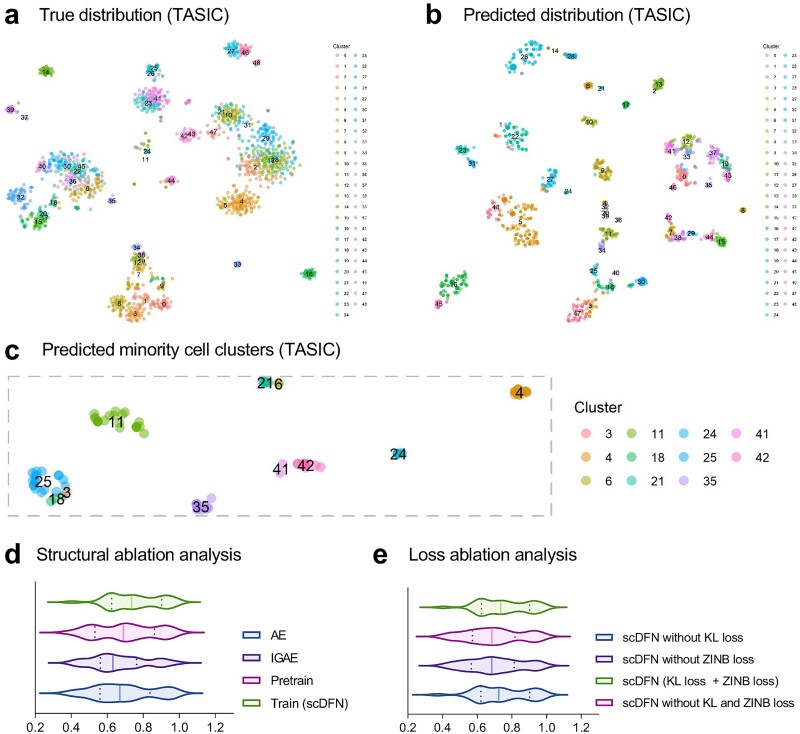
(a–c) The scDFN separated different cell types in the TASIC dataset: (a) Author’s noted labels on latent space; (b) the scDFN prediction of 49 cell types; (c) the projection of scDFN on small cell clusters. (d and e) Ablation studies: (d) comparison of ARI values for the 32 real scRNA-seq datasets with different modules [(i) AE only, (ii) IGAE only, (iii) the pre-training part after the fusion of AE and IGAE, and (iv) the fine-training part] and (e) comparison of ARI values for the 32 real scRNA-seq datasets with different losses.

The analysis of scRNA-seq datasets with a large number of cell clusters is challenging because unsupervised learning methods are affected by the number of cell clusters [54]. We found that scDFN can achieve better NMI and ARI performance on datasets with a large number of clusters (Supplementary Tables S2 and S3 available online at http://bib.oxfordjournals.org/). For example, for the Chen dataset (46 clusters) and the TASIC dataset (49 clusters), the NMI scores of scDFN were 0.7655 and 0.8178, and the ARI scores were 0.6420 and 0.5689, respectively. The high performance of scDFN occurred mainly because the newly constructed interactive potential space can capture the subtle differences in different cell clusters. For example, the TASIC dataset included 49 clusters, among which 9 clusters included less than 15 cells per cluster (Supplementary Table S7 available online at http://bib.oxfordjournals.org/). The original cell expression matrix of TASIC is visualized in Fig. 3a, which demonstrates that several clusters were mixed. After the interactive potential space was reconstructed in scDFN, most clusters were separated (Fig. 3b). To evaluate how scDFN distinguishes the rare cell clusters contained in TASIC, we selected the rare cell clusters to make the t-SNE projection and found that the cell clusters were well-separated in the potential space (Fig. 3c). In addition, the t-SNE projections of the three most advanced methods scMGCA, scDEFR and scTAG on the TASIC dataset are provided in Fig. S4 (available online at http://bib.oxfordjournals.org/).

### Superiority of information fusion

To assess the effectiveness of our proposed model at various stages and the contribution of each unit to the performance of scDFN, we conducted an ablation study containing (i) AE only, (ii) IGAE only, (iii) the pre-training part after the fusion of AE and IGAE, and (iv) the fine-training part. We evaluated the performance of different modules; the distribution of ARI values is shown in Fig. 3d; and the results of the NMI values and ARI values of different modules are shown in Supplementary Tables S8 and S9 (available online at http://bib.oxfordjournals.org/), respectively. The pre-training stage achieved an average NMI value of 0.7956 and an average ARI value of 0.6987 on 32 scRNA-seq datasets; these values were better than the average NMI and ARI values (0.7797 and 0.6912, respectively) obtained using only AE. Since we added triple self-supervised loss during the training stage, the average NMI and ARI values were further improved to 0.8161 and 0.7518, respectively. All results obtained indicated that the information fusion module can effectively extract the attribute information and topological information of single-cell data, thus improving the clustering performance.

### Analysis of the influence of different losses on the experimental performance

In our proposed scDFN, the decoder network used four loss functions to learn the latent representation, and thus it collaboratively optimized the ZINB reconstruction loss, the MSE reconstruction loss of AE, the MSE reconstruction loss of IGAE, and the KL triple self-supervised clustering loss. To examine the reasoning behind adopting ZINB loss and self-supervised triple clustering loss, we compared the performance of scDFN, scDFN without ZINB loss, scDFN without KL loss, and scDFN without both of them on 32 real scRNA-seq data. The distribution of ARI values ​​for different losses is shown in Fig. 3e, and the detailed results of NMI values and ARI values are shown in Supplementary Tables S10 and S11 (available online at http://bib.oxfordjournals.org/), respectively. We found that the performance of scDFN without KL and ZINB losses decreased considerably, with an average decrease in NMI of 0.0224 and an average decrease in ARI of 0.0542, whereas the performance of scDFN without KL loss decreased slightly, with an average decrease in NMI of 0.0032 and an average decrease in ARI of 0.0091. Using four types of losses, the NMI value improved on 14 datasets, while the ARI value improved on 16 datasets. Based on these findings, we concluded that ZINB loss and clustering loss in autoencoders positively affected the clustering performance of scDFER.

### Impact of cell cluster number and highly variable genes on scDFN

**Figure 4:**
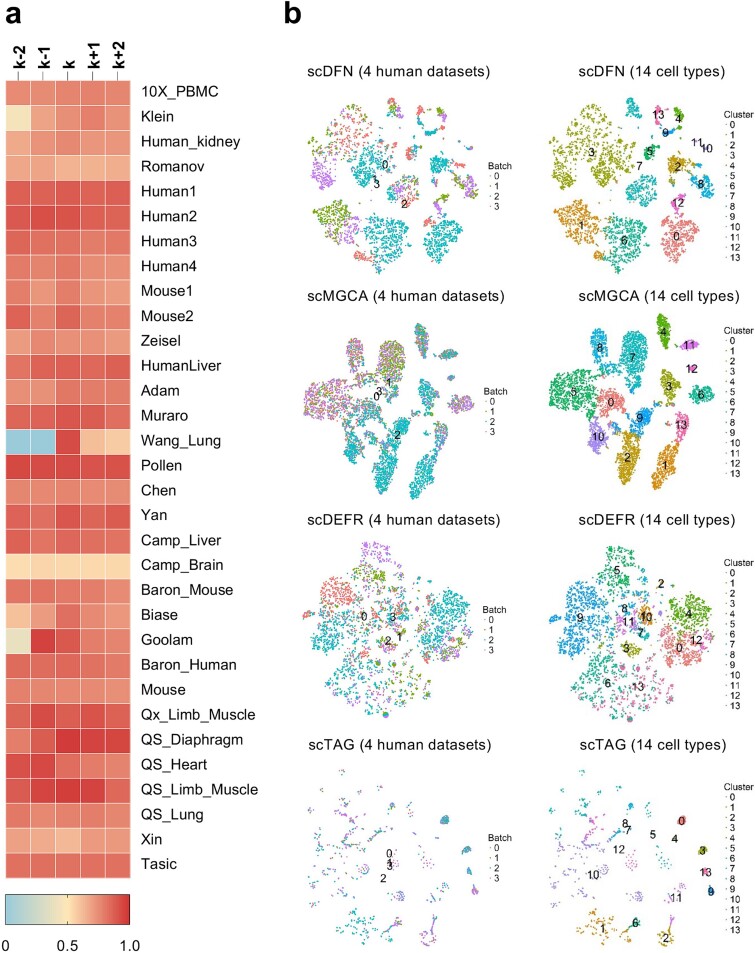
(a) The impact of different numbers of cell clusters on clustering performance (k represents the real number of cell clusters. (b) The t-SNE plots were used to compare the effectiveness of batch effect correction using four methods, including scDFN, scMGCA, scDEFR, and scTAG. The dataset used is a fusion of Human1 (Batch0), Human2 (Batch1), Human3 (Batch2), and Human4 (Batch3).

To determine whether the choice of the number of cell clusters affects the clustering performance of scDFN, we changed the number of clusters, i.e. we set the number of experimental clusters to $\left\{k-2,k-1,k,k+1,k+2\right\}$, where $k$ indicates the true number of clusters. The NMI results of scDFN on 32 datasets with different clusters are shown in Fig. 4a. The best NMI and ARI scores were obtained when $k$ was set as the true number of clusters, such as on 10X_PBMC [52], Romanov [43], Human [34], etc. Even when the best NMI value was not obtained under the actual number of clusters, changing the number of clusters had a negligible effect on the NMI value. For the datasets Wang_Lung ($K$ = 2), Biase ($K$ = 4), and Xin ($K$ = 4), the NMI values were more affected by interference, which occurred probably because the datasets contained few cell clusters. We observed that scDFN was not sensitive to the number of clusters (Fig. 4a). By comparing the average values, we found that changing the number of clusters had a greater impact on the ARI value than the NMI value (Supplementary Tables S12 and S13 available online at http://bib.oxfordjournals.org/). Overall, changing the number of clusters slightly affected the NMI and ARI values. The effects of different numbers of selected genes ($M$) on clustering performance are presented in Supplementary Tables S14 and S15 (available online at http://bib.oxfordjournals.org/). The results showed that scDFN had the best overall clustering performance for most datasets when *M* was 2000, compared to other conditions.

### scDFN can effectively eliminate the batch effect

Our scDFN method not only exhibited excellent performance in clustering but also showed its ability to effectively eliminate batch effects. Human1, Human2, Human3, and Human4 are single-cell RNA sequencing datasets from four human donor pancreatic cells. The number of cells in these datasets is 1937, 1724, 3605, and 1303, respectively. The number of genes is 20 215. Each dataset has 14 consistent cell types. These cells were derived from different batches [34]. As the cells were derived from different pancreatic tissues, there may be subtle differences between them related to batch effects. To evaluate the performance of scDFN in eliminating batch effects, we used R to integrate four datasets (Human1, Huamn2, Human3, and Human4). We obtained a combined dataset containing 8569 cells and 14 different cell clusters. We evaluated the ability of three state-of-the-art scRNA clustering methods, including scMGCA, scDEFR, and scTAG, to eliminate batch effects, determined by cluster visualization and batch correction results. The experimental results are shown in Fig. 4b. We found that scTAG could not completely separate some cell types, such as clusters 8, 10, and 12, scDEFR could not separate most cells, and scMGCA could effectively separate cells. Overall, scTAG and scDEFR performed poorly in eliminating batch effects. Both scDFN and scMGCA efficiently eliminated batch effects, demonstrated by clustering a mixture of cells of the same type but obtained from different batches. However, although scMGCA effectively eliminated batch effects, its clustering performance was not as good as that of scDFN because scDFN showed larger distances between clusters. To summarize, scDFN can mix cells of the same type but in different batches and accurately distinguish different cell types. The novelties of scDFN are the fusion of attribute and topology information, as well as the application of the quadruple joint loss function, which plays a major role in eliminating the batch effect.

### Finding marker genes

**Figure 5:**
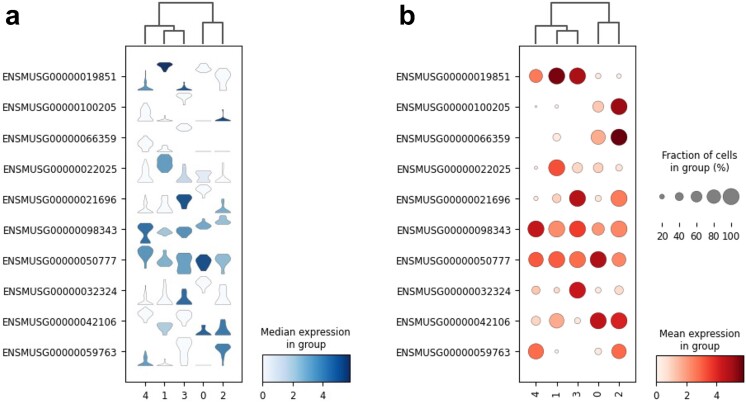
Visualization of the results of the Goolam marker genes. (a) A violin plot and (b) a bubble chart; the abscissa represents marker genes, and the ordinate represents cell clusters. The color depth of the violin plot reflects the degree of expression of the marker genes, while the shape of the violin plot shows the distribution of the expression of the marker genes in different cell types. The distribution of expression of the marker genes was different in different types of cells. The marker genes for each cluster were prominent.

Single-cell RNA sequencing data are often annotated using marker genes of the cell type, which can be predefined or defined de novo. Successful artificial interpretation requires high expression levels of closed cell clusters with distinct marker genes, and these genes are mainly concentrated in specific cell clusters. Finding marker genes related to cell clusters is a prerequisite for cell annotation, and the selection of marker genes strongly depends on the accuracy of the final results. In this study, we selected the Goolam dataset and used it to find the marker genes of each newborn [38]. For the Goolam dataset, we calculated the ranking of highly different genes in each cluster and only performed logistic regression analysis for validation (see Fig. S5 available online at http://bib.oxfordjournals.org/). Then, the top two genes from each cluster were selected for visualizing the results. The visualization results of Goolam are shown in Fig. 5. We used bubble plots and violin plots to display the expression of marker genes in each cell cluster. The marker genes for each cell cluster in the Goolam dataset are shown in Fig. 5. The results were visualized using the Python package Scanpy.

## Conclusion

In this study, a deep single-cell clustering model was developed through the fusion of AE and IGAE to identify cell populations in scRNA-seq datasets. First, high-dimensional scRNA-seq data were preprocessed, and the top highly variable genes were selected to eliminate redundant genes with low levels of expression that could interfere with the clustering results. These results indicated significant improvements in clustering performance. Next, AE and IGAE were combined through an information fusion mechanism to learn the attribute information and topological information of scRNA-seq data. Then, clustering was optimized using a quaternion loss function to identify cell types from the learned latent cell representations. To validate our model, we conducted a comprehensive study, in which we compared our model to other benchmark methods for cell type identification and characterization from different perspectives. We found that scDFN was better than the currently used methods. With the development of advanced high-throughput technologies for scRNA-seq and emerging cell atlases, we aim to investigate the performance of the proposed scDFN method on larger-scale scRNA-seq data in the future. One reason why scDFN did not consistently outperform other models was that it used hyperparameter settings uniformly across all datasets. However, this approach, in turn, decreased the problem of overfitting, which commonly occurs in methods trained on a specific dataset. This view was supported by the fact that the other five methods had lower performance rankings on average and showed larger differences than scDFN for the 32 datasets analyzed. In the future, we aim to expand the scope of scDFN and integrate different single-cell omics datasets, such as scATAC-seq and scMethyl-seq, to comprehensively understand cellular heterogeneity and epigenetic regulation.

**Key Points**

- We introduce a novel deep learning-based algorithm for single-cell clustering, designated scDFN through a fusion network strategy.
- We introduce a dual mechanism involving an autoencoder to extract attribute information and an improved graph autoencoder to capture topological nuances, integrated via a cross-network information fusion mechanism complemented by a triple self-supervision strategy.
- We confirm that scDFN substantially demonstrates robust multi-cluster dataset performance and exceptional resilience to batch effects.

## Supplementary Material

Supplement_tables-and-Figures_bbae486

Supplementary_information_FL_bbae486

## Data Availability

All datasets' information and access can be found in the Supplementary Information, while the source code for scFCN is accessible to the public at https://github.com/11051911/scDFN.
