# Supplementary material for: scDFN: enhancing single-cell RNA-seq clustering with deep fusion networks
Source: Brief Bioinform. 2024 Oct 5;25(6):bbae486. doi: 10.1093/bib/bbae486 (PMC11456827; doi:10.1093/bib/bbae486)
Supplement: Supplement_tables-and-Figures_bbae486 [file supplement_tables-and-figures_bbae486.docx]

Table S1. The statistics of 32 real scRNA-seq datasets.

| No. | dataset | organ | platform | cell types | cells | gene number |
| --- | --- | --- | --- | --- | --- | --- |
| 1 | 10X_PBMC | Human PBMC | 10X | 8 | 4271 | 16653 |
| 2 | Klein | Mouse embryo stem cells | inDrop | 4 | 2717 | 24175 |
| 3 | Human_kidney | kidney | 10X | 11 | 5685 | 25215 |
| 4 | Romanov | Mouse brain | SMARTer | 7 | 2881 | 24341 |
| 5 | Human1 | Human pancreas | inDrop | 14 | 1937 | 20125 |
| 6 | Human2 | Human pancreas | inDrop | 14 | 1724 | 20215 |
| 7 | Human3 | Human pancreas | inDrop | 14 | 3605 | 20125 |
| 8 | Human4 | Human pancreas | inDrop | 14 | 1303 | 20125 |
| 9 | Mouse1 | Mouse pancreas | inDrop | 13 | 822 | 14878 |
| 10 | Mouse2 | Mouse pancreas | inDrop | 13 | 1064 | 14878 |
| 11 | Zeisel | Mouse brain | Illumina HiSeq | 9 | 3005 | 19972 |
| 12 | HumanLiver | Liver | Unknown | 11 | 8444 | 5000 |
| 13 | Adam | Kidney | Drop-seq | 8 | 3660 | 23797 |
| 14 | Muraro | Pancreas | CEL-seq2 | 9 | 2122 | 19046 |
| 15 | Wang_Lung | Lung | 10X | 2 | 9519 | 14561 |
| 16 | Pollen | Human tissues | SMARTer | 11 | 301 | 21721 |
| 17 | Chen | Mouse brain | Drop-seq | 46 | 12089 | 23284 |
| 18 | Yan | Human embryo | Tang | 6 | 90 | 16383 |
| 19 | Camp_Liver | Liver | SMARTer | 7 | 777 | 19020 |
| 20 | Camp_Brain | Brain | SMARTer | 6 | 734 | 18927 |
| 21 | Baron_Mouse | Mouse pancreas | inDrop | 13 | 1886 | 20215 |
| 22 | Biase | Mus musculus | SMARTer | 4 | 56 | 25737 |
| 23 | Goolam | Mouse embryo | Smart-Seq2 | 5 | 124 | 41480 |
| 24 | Baron_Human | Human pancreas | inDrop | 14 | 8569 | 14878 |
| 25 | Mouse | Mus musculus | Microwell-seq | 16 | 2100 | 20670 |
| 26 | Qx_Limb_Muscle | Limb Muscle | 10X | 6 | 3909 | 23341 |
| 27 | QS_Diaphragm | Diaphragm | Smart-seq2 | 5 | 870 | 23341 |
| 28 | QS_Heart | Heart | Smart-seq2 | 8 | 4365 | 23341 |
| 29 | QS_Limb_Muscle | Limb Muscle | Smart-seq2 | 6 | 1090 | 23341 |
| 30 | QS_Lung | Lung | Smart-seq2 | 11 | 1676 | 23341 |
| 31 | Xin | Human pancreas | SMARTer | 4 | 1600 | 39851 |
| 32 | Tasic | Mouse visual cortex | SMARTer | 49 | 1727 | 24150 |

Table S2. Clustering performance comparison of the different clustering algorithms on 32 real scRNA-seq datasets measured by NMI.

| Dataset | scDFN | scDEFR | scDSSC | scTAG | scDeepCluster | scMGCA |
| --- | --- | --- | --- | --- | --- | --- |
| 10X_PBMC | 0.7665 | 0.7491 | 0.7440 | **0.7730** | 0.7704 | 0.7365 |
| Klein | 0.7386 | 0.6492 | **0.8503** | 0.7604 | 0.5604 | 0.6978 |
| Human_kidney | 0.7562 | 0.763 | 0.5984 | **0.8173** | 0.6214 | 0.7999 |
| Romanov | 0.6400 | 0.6831 | 0.7110 | 0.6070 | 0.5558 | **0.7341** |
| Human1 | 0.8803 | **0.8952** | 0.8146 | 0.6821 | 0.7944 | 0.7644 |
| Human2 | **0.9046** | 0.9018 | 0.8394 | 0.6799 | 0.7471 | 0.7112 |
| Human3 | 0.8189 | **0.8823** | 0.8461 | 0.7775 | 0.7915 | 0.8013 |
| Human4 | 0.7991 | 0.7545 | **0.8153** | 0.6563 | 0.7459 | 0.7463 |
| Mouse1 | **0.7833** | 0.7494 | 0.7191 | 0.6804 | 0.6816 | 0.7259 |
| Mouse2 | **0.8552** | 0.7617 | 0.8131 | 0.6363 | 0.6259 | 0.7754 |
| Zeisel | **0.7367** | 0.6987 | 0.634 | 0.5382 | 0.596 | 0.4687 |
| HumanLiver | **0.8697** | 0.8684 | 0.813 | 0.8275 | 0.7815 | 0.8573 |
| Adam | 0.8038 | **0.8781** | 0.8436 | 0.871 | 0.8238 | 0.8595 |
| Muraro | **0.8961** | 0.8903 | 0.8228 | 0.8406 | 0.7569 | 0.8379 |
| Wang_Lung | **0.9233** | 0.8684 | 0.001 | 0.8313 | 0.5263 | 0.6531 |
| Pollen | 0.9273 | 0.9135 | 0.8798 | 0.8925 | **0.9300** | 0.9197 |
| Chen | **0.7655** | 0.7599 | 0.5399 | 0.7643 | 0.7474 | 0.7651 |
| Yan | **0.8966** | 0.8929 | 0.7732 | 0.8046 | 0.7719 | 0.7922 |
| Camp_Liver | **0.8492** | 0.8307 | 0.7003 | 0.7767 | 0.7221 | 0.841 |
| Camp_Brain | 0.5388 | **0.5606** | 0.4003 | 0.5513 | 0.497 | 0.5536 |
| Baron_Mouse | 0.7969 | 0.7826 | **0.8462** | 0.675 | 0.7187 | 0.7337 |
| Biase | 0.8267 | 0.7737 | 0.495 | 0.1739 | **0.9232** | 0.2914 |
| Goolam | **0.8877** | 0.721 | 0.709 | 0.721 | 0.707 | 0.721 |
| Baron_Human | **0.8430** | 0.8421 | 0.5568 | 0.7729 | 0.759 | 0.581 |
| Mouse | **0.7596** | 0.7297 | 0.7203 | 0.697 | 0.7292 | 0.7102 |
| Qx_Limb_Muscle | 0.8731 | 0.9422 | 0.9619 | 0.9481 | 0.8769 | **0.9625** |
| QS_Diaphragm | **0.9693** | 0.9377 | 0.8498 | 0.9346 | 0.8027 | 0.9289 |
| QS_Heart | 0.8325 | 0.9008 | 0.8719 | 0.8857 | 0.6397 | **0.9068** |
| QS_Limb_Muscle | 0.9625 | **0.9637** | 0.897 | 0.9381 | 0.8153 | 0.9586 |
| QS_Lung | 0.7739 | **0.8133** | 0.7718 | 0.8083 | 0.697 | 0.7539 |
| Xin | 0.6221 | **0.6257** | 0.0071 | 0.5222 | 0.4694 | 0.4887 |
| Tasic | **0.8178** | 0.8166 | 0.5905 | 0.7369 | 0.5569 | 0.7073 |

Table S3. Clustering performance comparison of the different clustering algorithms on 32 real scRNA-seq datasets measured by ARI.

| Dataset | scDFN | scDEFR | scDSSC | scTAG | scDeepCluster | scMGCA |
| --- | --- | --- | --- | --- | --- | --- |
| 10X_PBMC | **0.7672** | 0.6751 | 0.6684 | 0.6698 | 0.7415 | 0.6704 |
| Klein | 0.756 | 0.6369 | **0.8134** | 0.7739 | 0.4559 | 0.6957 |
| Human_kidney | 0.6133 | 0.6355 | 0.2929 | **0.7228** | 0.4718 | 0.7215 |
| Romanov | 0.6328 | 0.6727 | 0.7207 | 0.4404 | 0.5114 | **0.7518** |
| Human1 | 0.8155 | **0.8358** | 0.7582 | 0.4821 | 0.5127 | 0.7022 |
| Human2 | **0.9129** | 0.912 | 0.8604 | 0.5197 | 0.486 | 0.6882 |
| Human3 | 0.6389 | **0.865** | 0.837 | 0.7402 | 0.622 | 0.7572 |
| Human4 | 0.6221 | 0.4958 | **0.8539** | 0.4271 | 0.4778 | 0.7367 |
| Mouse1 | **0.6209** | 0.4818 | 0.6045 | 0.4479 | 0.3917 | 0.4958 |
| Mouse2 | **0.8624** | 0.5289 | 0.8573 | 0.3688 | 0.3178 | 0.8321 |
| Zeisel | **0.7214** | 0.7212 | 0.6391 | 0.4894 | 0.4445 | 0.3075 |
| HumanLiver | **0.9202** | 0.8819 | 0.8447 | 0.841 | 0.6417 | 0.9018 |
| Adam | 0.7411 | **0.8851** | 0.8626 | 0.8747 | 0.7882 | 0.8635 |
| Muraro | **0.9351** | 0.9248 | 0.8737 | 0.8896 | 0.6103 | 0.8858 |
| Wang_Lung | **0.969** | 0.9387 | 0 | 0.9082 | 0.5176 | 0.7313 |
| Pollen | **0.9335** | 0.8665 | 0.8801 | 0.7941 | 0.8642 | 0.9267 |
| Chen | **0.642** | 0.6023 | 0.3309 | 0.5508 | 0.5396 | 0.5971 |
| Yan | **0.8783** | 0.8158 | 0.7467 | 0.7251 | 0.5957 | 0.6444 |
| Camp_Liver | 0.7296 | 0.6433 | 0.4834 | 0.6087 | 0.5367 | **0.7651** |
| Camp_Brain | 0.4053 | 0.4274 | 0.3728 | 0.4236 | 0.4003 | **0.4283** |
| Baron_Mouse | 0.6032 | 0.5532 | **0.8846** | 0.5522 | 0.4612 | 0.717 |
| Biase | 0.7252 | 0.5761 | 0.3098 | 0.0888 | **0.9217** | 0.1953 |
| Goolam | **0.9097** | 0.5439 | 0.763 | 0.5439 | 0.5432 | 0.5439 |
| Baron_Human | **0.7906** | 0.6798 | 0.3156 | 0.7518 | 0.5728 | 0.334 |
| Mouse | 0.5907 | 0.5447 | **0.6161** | 0.4668 | 0.5162 | 0.5043 |
| Qx_Limb_Muscle | 0.8023 | 0.9542 | **0.9829** | 0.9581 | 0.8502 | 0.9811 |
| QS_Diaphragm | **0.9813** | 0.9545 | 0.9103 | 0.9628 | 0.6561 | 0.9559 |
| QS_Heart | 0.6772 | 0.9448 | 0.9235 | 0.9371 | 0.3873 | **0.9516** |
| QS_Limb_Muscle | 0.9786 | **0.9802** | 0.9215 | 0.967 | 0.6925 | 0.9769 |
| QS_Lung | 0.6156 | 0.7002 | **0.8639** | 0.6526 | 0.4101 | 0.6969 |
| Xin | **0.6968** | 0.691 | 0.0058 | 0.6893 | 0.2666 | 0.651 |
| Tasic | **0.5689** | 0.5579 | 0.2498 | 0.4539 | 0.2362 | 0.391 |

Table S4. Clustering performance comparison of the different clustering algorithms on 32 real scRNA-seq datasets measured by ASW.

| Dataset | scDFN | scDEFR | scDSSC | scTAG | scDeepCluster | scMGCA |
| --- | --- | --- | --- | --- | --- | --- |
| 10X_PBMC | 0.5949 | 0.3179 | 0.4021 | 0.9305 | 0.8266 | 0.7633 |
| Klein | 0.5828 | 0.6436 | 0.378 | 0.9465 | 0.7283 | 0.4544 |
| Human_kidney | 0.3426 | 0.1663 | 0.2516 | 0.9309 | 0.8356 | 0.7598 |
| Romanov | 0.3833 | 0.2734 | 0.3356 | 0.8899 | 0.7889 | 0.5416 |
| Human1 | 0.6139 | 0.4076 | 0.4109 | 0.7587 | 0.8201 | 0.7628 |
| Human2 | 0.7293 | 0.4277 | 0.4105 | 0.7889 | 0.8822 | 0.8061 |
| Human3 | 0.4931 | 0.3314 | 0.3131 | 0.8236 | 0.881 | 0.7625 |
| Human4 | 0.4396 | 0.2241 | 0.3006 | 0.7841 | 0.8337 | 0.7732 |
| Mouse1 | 0.5633 | 0.3148 | 0.1483 | 0.882 | 0.7948 | 0.7704 |
| Mouse2 | 0.5498 | 0.255 | 0.4228 | 0.6707 | 0.7306 | 0.8248 |
| Zeisel | 0.4068 | 0.1799 | 0.3186 | 0.8838 | 0.8584 | 0.7539 |
| HumanLiver | 0.393 | 0.2932 | 0.1706 | 0.9503 | 0.7875 | 0.7841 |
| Adam | 0.5476 | 0.3162 | 0.2919 | 0.9199 | 0.6880 | 0.6495 |
| Muraro | 0.6223 | 0.5638 | 0.5306 | 0.9181 | 0.6334 | 0.5584 |
| Wang_Lung | 0.6544 | 0.4552 | 0.4857 | 0.9653 | 0.5216 | 0.7267 |
| Pollen | 0.7803 | 0.7559 | 0.3176 | 0.9773 | 0.7338 | 0.7286 |
| Chen | 0.5065 | 0.1622 | 0 | 0.6827 | 0.0836 | 0.4604 |
| Yan | 0.8844 | 0.7353 | 0.224 | 0.7251 | 0.5294 | 0.8442 |
| Camp_Liver | 0.7175 | 0.6688 | 0.3344 | 0.9625 | 0.4636 | 0.6177 |
| Camp_Brain | 0.4733 | 0.0783 | 0.2507 | 0.9812 | 0.2435 | 0.2201 |
| Baron_Mouse | 0.6246 | 0.2143 | 0.3643 | 0.8297 | 0.8707 | 0.7745 |
| Biase | 0.6246 | 0.4255 | 0.0073 | 0.7715 | 0.2716 | 0.7552 |
| Goolam | 0.8443 | 0.8146 | 0.2594 | 0.9582 | 0.3547 | 0.8617 |
| Baron_Human | 0.5549 | 0.2064 | 0.13155 | 0.787 | 0.943 | 0.6671 |
| Mouse | 0.3674 | 0.2672 | 0.2008 | 0.8237 | 0.8409 | 0.7366 |
| Qx_Limb_Muscle | 0.5620 | 0.3579 | 0.4144 | 0.9685 | 0.7966 | 0.8337 |
| QS_Diaphragm | 0.7869 | 0.7263 | 0..5242 | 0.9604 | 0.722 | 0.8458 |
| QS_Heart | 0.474 | 0.5856 | 0.425 | 0.9527 | 0.5336 | 0.6643 |
| QS_Limb_Muscle | 0.6696 | 0.7263 | 0.4867 | 0.9655 | 0.7324 | 0.8207 |
| QS_Lung | 0.3564 | 0.4249 | 0.1977 | 0.971 | 0.4409 | 0.5414 |
| Xin | 0.5583 | 0.4522 | 0.2396 | 0.9496 | 0.7919 | 0.6426 |
| Tasic | 0.5686 | 0.1893 | 0.0619 | 0.7506 | 0.5573 | 0.7096 |

Table S5. Clustering performance comparison of the different clustering algorithms on 32 real scRNA-seq datasets measured by cLISI.

| Dataset | scDFN | scDEFR | scDSSC | scTAG | scDeepCluster | scMGCA |
| --- | --- | --- | --- | --- | --- | --- |
| 10X_PBMC | 1 | 1 | 1 | 1 | 1 | 1 |
| Klein | 1 | 1 | 1 | 1 | 1 | 1 |
| Human_kidney | 1 | 1 | 0.9978 | 1 | 1 | 1 |
| Romanov | 1 | 1 | 1 | 1 | 1 | 1 |
| Human1 | 1 | 1 | 1 | 1 | 1 | 1 |
| Human2 | 1 | 1 | 1 | 1 | 1 | 1 |
| Human3 | 0.9947 | 1 | 0.9983 | 1 | 1 | 1 |
| Human4 | 0.9676 | 0.9453 | 0.9965 | 1 | 1 | 1 |
| Mouse1 | 0.9516 | 0.948 | 0.9375 | 0.9694 | 0.9743 | 1 |
| Mouse2 | 0.9962 | 0.9582 | 1 | 0.9981 | 1 | 1 |
| Zeisel | 1 | 0.9972 | 0.9972 | 1 | 1 | 1 |
| HumanLiver | 1 | 1 | 0.9358 | 1 | 1 | 1 |
| Adam | 1 | 1 | 0.9968 | 1 | 1 | 1 |
| Muraro | 1 | 1 | 1 | 1 | 1 | 1 |
| Wang_Lung | 1 | 1 | 1 | 1 | 1 | 1 |
| Pollen | 1 | 0.7672 | 0.8202 | 0.7989 | 0.7553 | 1 |
| Chen | 1 | 0.9968 | 0.9571 | 1 | 1 | 1 |
| Yan | 1 | 1 | 0.9561 | 1 | 1 | 1 |
| Camp_Liver | 1 | 1 | 1 | 1 | 1 | 1 |
| Camp_Brain | 0.9838 | 0.8361 | 1 | 1 | 1 | 1 |
| Baron_Mouse | 1 | 0.9943 | 1 | 1 | 1 | 1 |
| Biase | 1 | 1 | 0.7586 | 1 | 1 | 1 |
| Goolam | 1 | 1 | 1 | 1 | 1 | 1 |
| Baron_Human | 1 | 1 | 0.9443 | 1 | 1 | 1 |
| Mouse | 1 | 0.9705 | 1 | 1 | 1 | 1 |
| Qx_Limb_Muscle | 1 | 1 | 1 | 1 | 1 | 1 |
| QS_Diaphragm | 1 | 1 | 1 | 1 | 1 | 1 |
| QS_Heart | 1 | 1 | 1 | 1 | 1 | 1 |
| QS_Limb_Muscle | 1 | 1 | 1 | 1 | 1 | 1 |
| QS_Lung | 1 | 1 | 0.9856 | 1 | 1 | 1 |
| Xin | 1 | 1 | 1 | 1 | 1 | 1 |
| Tasic | 1 | 0.9436 | 1 | 0.9694 | 1 | 1 |

Table S6. Running time comparison of the different clustering algorithms on 32 real scRNA-seq datasets.

| Dataset | scDFN | scDEFR | scDSSC | scTAG | scDeepCluster | scMGCA |
| --- | --- | --- | --- | --- | --- | --- |
| 10X_PBMC | 2.9373 | 26.005 | 3.0475 | 1.5864 | 1.9507 | 4.2533 |
| Klein | 2.7045 | 58.971 | 1.8296 | 0.9156 | 2.7281 | 2.1699 |
| Human_kidney | 3.6987 | 125.955 | 4.174 | 2.0479 | 4.4969 | 5.5068 |
| Romanov | 2.844 | 72.4325 | 2.177 | 0.9754 | 2.0698 | 2.3534 |
| Human1 | 2.5998 | 90.322 | 1.7746 | 0.6827 | 1.0546 | 2.1637 |
| Human2 | 2.5182 | 53.8235 | 1.2621 | 0.6227 | 1.1519 | 1.3581 |
| Human3 | 2.9274 | 75.8375 | 2.9221 | 1.2034 | 2.238 | 3.0333 |
| Human4 | 2.3844 | 38.671 | 0.973 | 0.5462 | 0.7626 | 1.0889 |
| Mouse1 | 2.2038 | 7.609 | 0.6522 | 0.4819 | 0.4586 | 0.7394 |
| Mouse2 | 2.2458 | 8.5535 | 0.7511 | 0.4999 | 0.5306 | 1.1773 |
| Zeisel | 2.9415 | 20.6655 | 2.1468 | 1.0113 | 3.0188 | 2.4426 |
| HumanLiver | 5.0115 | 102.1685 | 8.1857 | 4.0594 | 1.1674 | 9.947 |
| Adam | 2.7426 | 118.9583333 | 4.69 | 1.2205 | 2.2977 | 3.0021 |
| Muraro | 2.5377 | 13 | 3.0355 | 0.7468 | 1.1813 | 1.7434 |
| Wang_Lung | 11.3049 | 236.65 | 12.9269 | 5.2797 | 3.1556 | 12.6932 |
| Pollen | 1.9137 | 6.136 | 0.8624 | 0.4442 | 0.2215 | 0.3155 |
| Chen | 14.0256 | 235.658 | 19.9135 | 5.359 | 9.1659 | 19.5449 |
| Yan | 1.8816 | 5.82675 | 0.139 | 1.1185 | 0.0861 | 0.1759 |
| Camp_Liver | 2.1357 | 7.3195 | 0.8057 | 0.5405 | 0.4924 | 0.7766 |
| Camp_Brain | 2.127 | 6.794 | 0.5592 | 0.5551 | 0.4938 | 0.841 |
| Baron_Mouse | 2.5296 | 11.5245 | 1.3069 | 0.637 | 1.2217 | 1.9607 |
| Biase | 1.8615 | 5.681 | 0.1225 | 1.0486 | 0.0936 | 0.1549 |
| Goolam | 1.8822 | 5.8585 | 0.3058 | 1.2291 | 0.1364 | 0.1923 |
| Baron_Human | 7.6212 | 85.5785 | 9.1821 | 4.4236 | 8.6381 | 11.0914 |
| Mouse | 2.2788 | 12.656 | 1.7822 | 0.7797 | 1.7502 | 2.2512 |
| Qx_Limb_Muscle | 2.6982 | 23.988 | 6.5104 | 1.2621 | 1.5836 | 3.0071 |
| QS_Diaphragm | 2.0085 | 7.2725 | 1.1712 | 2.5096 | 0.4135 | 0.7799 |
| QS_Heart | 4.2681 | 33.69 | 2.9432 | 1.4285 | 5.2651 | 5.3399 |
| QS_Limb_Muscle | 2.1693 | 7.74 | 0.6542 | 0.4855 | 0.5539 | 0.9212 |
| QS_Lung | 2.4057 | 26.7525 | 1.0108 | 0.5919 | 2.2496 | 1.3232 |
| Xin | 2.4195 | 9.3375 | 1.2821 | 1.7659 | 0.8872 | 1.8099 |
| Tasic | 3.363 | 13.1555 | 1.1133 | 0.7173 | 1.2202 | 1.541 |

Table S7. The number of cells on the Tasic dataset and the correspondence between real cell type and cell number.

| Cell type | Cluster number | Cell number |
| --- | --- | --- |
| Vip Mybpc1 | 0 | 29 |
| Vip Parm1 | 1 | 45 |
| L4 Ctxn3 | 2 | 79 |
| Vip Chat | 3 | 48 |
| L2/3 Ptgs2 | 4 | 96 |
| L2 Ngb | 5 | 21 |
| Pvalb Gpx3 | 6 | 63 |
| Ndnf Cxcl14 | 7 | 35 |
| Vip Gpc3 | 8 | 50 |
| Vip Sncg | 9 | 14 |
| L5a Batf3 | 10 | 66 |
| Endo Myl9 | 11 | 15 |
| Ndnf Car4 | 12 | 31 |
| L4 Scnn1a | 13 | 99 |
| Astro Gja1 | 14 | 43 |
| Sst Myh8 | 15 | 41 |
| Sst Chodl | 16 | 41 |
| Sst Th | 17 | 19 |
| Sst Tacstd2 | 18 | 14 |
| Sncg | 19 | 9 |
| Sst Cdk6 | 20 | 19 |
| L5a Pde1c | 21 | 19 |
| Pvalb Wt1 | 22 | 58 |
| L6a Mgp | 23 | 53 |
| Endo Tbc1d4 | 24 | 14 |
| L6b Serpinb11 | 25 | 20 |
| L6b Rgs12 | 26 | 15 |
| L5b Cdh13 | 27 | 42 |
| L4 Arf5 | 28 | 44 |
| L5a Hsd11b1 | 29 | 53 |
| Pvalb Tacr3 | 30 | 71 |
| L5a Tcerg1l | 31 | 35 |
| Sst Cbln4 | 32 | 68 |
| Micro Ctss | 33 | 22 |
| Igtp | 34 | 10 |
| Pvalb Cpne5 | 35 | 14 |
| Pvalb Rspo2 | 36 | 31 |
| Oligo 96_Rik | 37 | 8 |
| Smad3 | 38 | 13 |
| Oligo Opalin | 39 | 30 |
| Pvalb Tpbg | 40 | 18 |
| L6a Sla | 41 | 74 |
| L6a Syt17 | 42 | 15 |
| L6a Car12 | 43 | 22 |
| OPC Pdgfra | 44 | 22 |
| Pvalb Obox3 | 45 | 20 |
| L5b Tph2 | 46 | 32 |
| L5 Ucma | 47 | 16 |
| L5 Chrna6 | 48 | 11 |

Table S8. Clustering result comparison of scDFN in different module ((i) only AE; (ii) only IGAE; (iii) the pre-training part after the fusion of AE and IGAE; (iv) the training part.) measured by NMI.

| Dataset | only AE | only IGAE | pretrain | train |
| --- | --- | --- | --- | --- |
| 10X_PBMC | 0.7435 | 0.7329 | 0.7557 | 0.7665 |
| Klein | 0.7396 | 0.74 | 0.733 | 0.7386 |
| Human_kidney | 0.7121 | 0.6747 | 0.7205 | 0.7562 |
| Romanov | 0.6832 | 0.6124 | 0.6889 | 0.64 |
| Human1 | 0.8463 | 0.8409 | 0.8692 | 0.8803 |
| Human2 | 0.8868 | 0.8397 | 0.8843 | 0.9046 |
| Human3 | 0.7959 | 0.8092 | 0.7997 | 0.8189 |
| Human4 | 0.7485 | 0.7761 | 0.7645 | 0.7991 |
| Mouse1 | 0.7455 | 0.7427 | 0.7284 | 0.7833 |
| Mouse2 | 0.7474 | 0.7751 | 0.7675 | 0.8552 |
| Zeisel | 0.6643 | 0.7101 | 0.7516 | 0.7367 |
| HumanLiver | 0.81 | 0.7854 | 0.8416 | 0.8697 |
| Adam | 0.7635 | 0.7444 | 0.7853 | 0.8038 |
| Muraro | 0.8859 | 0.8547 | 0.8909 | 0.8961 |
| Wang_Lung | 0.9217 | 0.8677 | 0.891 | 0.9233 |
| Pollen | 0.9383 | 0.9163 | 0.9256 | 0.9273 |
| Chen | 0.727 | 0.7389 | 0.7709 | 0.7655 |
| Yan | 0.772 | 0.8696 | 0.8966 | 0.8966 |
| Camp_Liver | 0.8272 | 0.8686 | 0.8516 | 0.8492 |
| Camp_Brain | 0.5099 | 0.5304 | 0.5239 | 0.5388 |
| Baron_Mouse | 0.7721 | 0.7664 | 0.7635 | 0.7969 |
| Biase | 0.62 | 0.5219 | 0.6803 | 0.8267 |
| Goolam | 0.8285 | 0.6968 | 0.8333 | 0.8877 |
| Baron_Human | 0.7884 | 0.7743 | 0.8003 | 0.843 |
| Mouse | 0.738 | 0.7584 | 0.7412 | 0.7596 |
| Qx_Limb_Muscle | 0.8387 | 0.9662 | 0.8626 | 0.8731 |
| QS_Diaphragm | 0.9504 | 0.9402 | 0.9448 | 0.9693 |
| QS_Heart | 0.8474 | 0.262 | 0.8369 | 0.8325 |
| QS_Limb_Muscle | 0.9395 | 0.835 | 0.9656 | 0.9625 |
| QS_Lung | 0.7598 | 0.7378 | 0.7354 | 0.7739 |
| Xin | 0.6013 | 0.6193 | 0.6254 | 0.6221 |
| Tasic | 0.7966 | 0.8242 | 0.8276 | 0.8178 |
| Average | **0.7797** | **0.7541** | **0.7956** | **0.8161** |

Table S9. Clustering result comparison of scDFN in different module ((i) only AE; (ii) only IGAE; (iii) the pre-training part after the fusion of AE and IGAE; (iv) the training part.) measured by ARI.

| Dataset | only AE | only IGAE | pretrain | train |
| --- | --- | --- | --- | --- |
| 10X_PBMC | 0.6764 | 0.6337 | 0.6881 | 0.7672 |
| Klein | 0.7669 | 0.7555 | 0.7497 | 0.756 |
| Human_kidney | 0.5337 | 0.5572 | 0.5069 | 0.6133 |
| Romanov | 0.7328 | 0.6183 | 0.7474 | 0.6328 |
| Human1 | 0.7886 | 0.7651 | 0.8151 | 0.8155 |
| Human2 | 0.9079 | 0.7403 | 0.9032 | 0.9129 |
| Human3 | 0.6044 | 0.6289 | 0.5829 | 0.6389 |
| Human4 | 0.4888 | 0.5563 | 0.5149 | 0.6221 |
| Mouse1 | 0.5649 | 0.4496 | 0.4421 | 0.6209 |
| Mouse2 | 0.5421 | 0.5828 | 0.5588 | 0.8624 |
| Zeisel | 0.6154 | 0.6849 | 0.7888 | 0.7214 |
| HumanLiver | 0.8531 | 0.6213 | 0.8829 | 0.9202 |
| Adam | 0.7117 | 0.6235 | 0.7099 | 0.7411 |
| Muraro | 0.9246 | 0.8964 | 0.9255 | 0.9351 |
| Wang_Lung | 0.9677 | 0.937 | 0.9522 | 0.969 |
| Pollen | 0.9422 | 0.8747 | 0.9244 | 0.9335 |
| Chen | 0.4324 | 0.7572 | 0.6775 | 0.642 |
| Yan | 0.6631 | 0.8783 | 0.8783 | 0.8783 |
| Camp_Liver | 0.7109 | 0.7896 | 0.732 | 0.7296 |
| Camp_Brain | 0.3836 | 0.4136 | 0.3855 | 0.4053 |
| Baron_Mouse | 0.6265 | 0.481 | 0.4741 | 0.6032 |
| Biase | 0.4387 | 0.4305 | 0.4934 | 0.7252 |
| Goolam | 0.6566 | 0.5335 | 0.6784 | 0.9097 |
| Baron_Human | 0.6235 | 0.5775 | 0.6374 | 0.7906 |
| Mouse | 0.5579 | 0.5766 | 0.5228 | 0.5907 |
| Qx_Limb_Muscle | 0.751 | 0.9822 | 0.7946 | 0.8023 |
| QS_Diaphragm | 0.9717 | 0.9041 | 0.958 | 0.9813 |
| QS_Heart | 0.8749 | 0.6943 | 0.6886 | 0.6772 |
| QS_Limb_Muscle | 0.9708 | 0.6808 | 0.9788 | 0.9786 |
| QS_Lung | 0.5838 | 0.4924 | 0.4991 | 0.6156 |
| Xin | 0.7283 | 0.6945 | 0.6911 | 0.6968 |
| Tasic | 0.524 | 0.5862 | 0.5756 | 0.5689 |
| Average | **0.6912** | **0.6687** | **0.6987** | **0.7518** |

Table S10. Clustering result comparison of scDFN with or without loss measured by NMI.

| Dataset | WithoutKL loss | without ZINB loss | without KL loss and ZINB loss | scDFN |
| --- | --- | --- | --- | --- |
| 10X_PBMC | 0.7687 | 0.7294 | 0.7555 | 0.7665 |
| Klein | 0.7286 | 0.7466 | 0.7404 | 0.7386 |
| Human_kidney | 0.7476 | 0.7198 | 0.7104 | **0.7562** |
| Romanov | 0.6386 | 0.7 | 0.6985 | 0.64 |
| Human1 | 0.8785 | 0.8676 | 0.8675 | **0.8803** |
| Human2 | 0.9 | 0.8801 | 0.8842 | **0.9046** |
| Human3 | 0.8135 | 0.8255 | 0.8083 | **0.8189** |
| Human4 | 0.7997 | 0.769 | 0.7513 | 0.7991 |
| Mouse1 | 0.7628 | 0.7307 | 0.7286 | **0.7833** |
| Mouse2 | 0.8548 | 0.7717 | 0.7692 | **0.8552** |
| Zeisel | 0.7349 | 0.7463 | 0.7328 | 0.7367 |
| HumanLiver | 0.8815 | 0.8384 | 0.8389 | 0.8697 |
| Adam | 0.8058 | 0.8 | 0.7966 | 0.8038 |
| Muraro | 0.8917 | 0.8882 | 0.8891 | **0.8961** |
| Wang_Lung | 0.9191 | 0.889 | 0.8905 | **0.9233** |
| Pollen | 0.9208 | 0.9264 | 0.9243 | **0.9273** |
| Chen | 0.7646 | 0.7766 | 0.7621 | 0.7655 |
| Yan | 0.8966 | 0.7926 | 0.7926 | **0.8966** |
| Camp_Liver | 0.8431 | 0.8503 | 0.8523 | 0.8492 |
| Camp_Brain | 0.5179 | 0.5531 | 0.5396 | 0.5388 |
| Baron_Mouse | 0.7941 | 0.7618 | 0.7647 | **0.7969** |
| Biase | 0.8267 | 0.6831 | 0.6831 | **0.8267** |
| Goolam | 0.9184 | 0.8346 | 0.8346 | 0.8877 |
| Baron_Human | 0.8233 | 0.7952 | 0.8154 | **0.843** |
| Mouse | 0.7408 | 0.7579 | 0.7602 | 0.7596 |
| Qx_Limb_Muscle | 0.8593 | 0.876 | 0.8745 | 0.8731 |
| QS_Diaphragm | 0.9657 | 0.9429 | 0.9448 | **0.9693** |
| QS_Heart | 0.8343 | 0.8317 | 0.8339 | 0.8325 |
| QS_Limb_Muscle | 0.9595 | 0.9644 | 0.9717 | 0.9625 |
| QS_Lung | 0.7772 | 0.7188 | 0.7391 | 0.7739 |
| Xin | 0.6225 | 0.6254 | 0.6168 | 0.6221 |
| Tasic | 0.8223 | 0.8288 | 0.8269 | 0.8178 |
| Average | **0.8129** | **0.7944** | **0.7937** | **0.8161** |

Table S11. Clustering result comparison of scDFN with or without loss measured by ARI.

| Dataset | Without KL loss | without ZINB loss | without KL loss and ZINB loss | scDFN |
| --- | --- | --- | --- | --- |
| 10X_PBMC | 0.7697 | 0.6636 | 0.6682 | 0.7672 |
| Klein | 0.7475 | 0.7629 | 0.758 | 0.756 |
| Human_kidney | 0.5997 | 0.537 | 0.5236 | **0.6133** |
| Romanov | 0.6329 | 0.7578 | 0.759 | 0.6328 |
| Human1 | 0.8113 | 0.8156 | 0.8216 | 0.8155 |
| Human2 | 0.9101 | 0.8496 | 0.9031 | **0.9129** |
| Human3 | 0.6218 | 0.637 | 0.6151 | **0.6389** |
| Human4 | 0.6212 | 0.5272 | 0.4568 | **0.6221** |
| Mouse1 | 0.5967 | 0.4495 | 0.4432 | **0.6209** |
| Mouse2 | 0.8588 | 0.5583 | 0.5598 | **0.8624** |
| Zeisel | 0.7231 | 0.7852 | 0.7422 | 0.7214 |
| HumanLiver | 0.9253 | 0.8801 | 0.8801 | 0.9202 |
| Adam | 0.7434 | 0.741 | 0.7382 | 0.7411 |
| Muraro | 0.9288 | 0.9242 | 0.9205 | **0.9351** |
| Wang_Lung | 0.9672 | 0.9508 | 0.9517 | **0.969** |
| Pollen | 0.9157 | 0.9242 | 0.9229 | **0.9335** |
| Chen | 0.6383 | 0.6712 | 0.6176 | 0.642 |
| Yan | 0.8783 | 0.6764 | 0.6764 | **0.8783** |
| Camp_Liver | 0.7218 | 0.7305 | 0.7328 | 0.7296 |
| Camp_Brain | 0.3712 | 0.412 | 0.4007 | 0.4053 |
| Baron_Mouse | 0.5986 | 0.4735 | 0.4751 | **0.6032** |
| Biase | 0.7252 | 0.6 | 0.6 | **0.7252** |
| Goolam | 0.9633 | 0.6842 | 0.6842 | 0.9097 |
| Baron_Human | 0.6417 | 0.635 | 0.6493 | **0.7906** |
| Mouse | 0.5329 | 0.5555 | 0.5645 | **0.5907** |
| Qx_Limb_Muscle | 0.7881 | 0.8073 | 0.8067 | 0.8023 |
| QS_Diaphragm | 0.9783 | 0.9569 | 0.958 | **0.9813** |
| QS_Heart | 0.692 | 0.684 | 0.6848 | 0.6772 |
| QS_Limb_Muscle | 0.976 | 0.9805 | 0.9846 | 0.9786 |
| QS_Lung | 0.6121 | 0.4892 | 0.5332 | **0.6156** |
| Xin | 0.702 | 0.6991 | 0.6953 | 0.6968 |
| Tasic | 0.5724 | 0.5907 | 0.5964 | 0.5689 |
| Average | **0.7427** | **0.7003** | **0.6976** | **0.7518** |

Table S12. The impact of different numbers of a cell clusters by NMI (k is true cluster number).

| Dataset | k-2 | k-1 | k | k+1 | k+2 |
| --- | --- | --- | --- | --- | --- |
| 10X_PBMC | 0.7487 | 0.7468 | 0.7665 | **0.7745** | 0.7605 |
| Klein | 0.4423 | 0.6812 | 0.7386 | **0.7791** | 0.7313 |
| Human_kidney | 0.6647 | 0.7274 | **0.7562** | 0.7197 | 0.72 |
| Romanov | 0.674 | **0.6775** | 0.64 | 0.6432 | 0.6532 |
| Human1 | 0.8701 | 0.863 | **0.8803** | 0.8613 | 0.8703 |
| Human2 | 0.8871 | **0.916** | 0.9046 | 0.87 | 0.8588 |
| Human3 | **0.8532** | 0.8216 | 0.8189 | 0.7885 | 0.8 |
| Human4 | 0.7954 | 0.771 | **0.7991** | 0.7437 | 0.7373 |
| Mouse1 | **0.785** | 0.72 | 0.7833 | 0.7222 | 0.7031 |
| Mouse2 | **0.8618** | 0.7772 | 0.8552 | 0.7773 | 0.7749 |
| Zeisel | 0.7042 | 0.7589 | 0.7367 | **0.7368** | 0.7096 |
| HumanLiver | 0.8123 | 0.861 | **0.8697** | 0.8477 | 0.8654 |
| Adam | 0.7377 | 0.7323 | **0.8038** | 0.7875 | 0.7891 |
| Muraro | 0.8521 | 0.8603 | **0.8961** | 0.8775 | 0.8248 |
| Wang_Lung | 0 | 0 | **0.9233** | 0.6095 | 0.5744 |
| Pollen | **0.9335** | 0.9277 | 0.9273 | 0.9021 | 0.9085 |
| Chen | 0.7606 | 0.7595 | **0.7655** | 0.75 | 0.7504 |
| Yan | 0.86 | 0.8267 | **0.8966** | 0.8547 | 0.8801 |
| Camp_Liver | **0.864** | 0.8122 | 0.8492 | 0.8243 | 0.8166 |
| Camp_Brain | 0.5225 | **0.5445** | 0.5388 | 0.5359 | 0.5405 |
| Baron_Mouse | 0.8069 | **0.813** | 0.7969 | 0.785 | 0.7486 |
| Biase | 0.6019 | 0.7065 | **0.8267** | 0.7524 | 0.7177 |
| Goolam | 0.3866 | **0.9508** | 0.8877 | 0.783 | 0.7652 |
| Baron_Human | 0.8356 | 0.8335 | 0.843 | 0.8191 | 0.7935 |
| Mouse | **0.7763** | 0.7608 | 0.7596 | 0.7528 | 0.7386 |
| Qx_Limb_Muscle | 0.8554 | **0.9231** | 0.8731 | 0.9042 | 0.8628 |
| QS_Diaphragm | 0.7829 | 0.8761 | **0.9693** | 0.9462 | 0.9415 |
| QS_Heart | 0.9125 | **0.9343** | 0.8325 | 0.7916 | 0.7706 |
| QS_Limb_Muscle | 0.883 | 0.9426 | **0.9625** | 0.9481 | 0.8477 |
| QS_Lung | **0.7993** | 0.7592 | 0.7739 | 0.7847 | 0.7641 |
| Xin | 0.6899 | 0.6617 | 0.6221 | 0.7073 | **0.7115** |
| Tasic | 0.8206 | **0.8236** | 0.8178 | 0.8221 | 0.8219 |
| Average | **0.7431** | **0.7741** | **0.8161** | **0.7876** | **0.7735** |

Table S13. The impact of different numbers of a cell clusters by ARI (k is true cluster number).

| Dataset | k-2 | k-1 | k | k+1 | k+2 |
| --- | --- | --- | --- | --- | --- |
| 10X_PBMC | 0.6642 | 0.6671 | **0.7672** | 0.7665 | 0.7672 |
| Klein | 0.3764 | 0.6726 | 0.756 | **0.8072** | 0.7435 |
| Human_kidney | 0.4366 | 0.5544 | **0.6133** | 0.5322 | 0.5218 |
| Romanov | 0.7365 | **0.7375** | 0.6328 | 0.637 | 0.6445 |
| Human1 | **0.8262** | 0.82 | 0.8155 | 0.8222 | 0.8251 |
| Human2 | 0.9048 | **0.9194** | 0.9129 | 0.8439 | 0.8351 |
| Human3 | **0.7224** | 0.6354 | 0.6389 | 0.6058 | 0.6051 |
| Human4 | 0.5833 | 0.521 | **0.6221** | 0.4436 | 0.4373 |
| Mouse1 | **0.6223** | 0.4403 | 0.6209 | 0.4432 | 0.3996 |
| Mouse2 | 0.8517 | 0.5867 | **0.8624** | 0.5607 | 0.5608 |
| Zeisel | 0.6629 | **0.7973** | 0.7214 | 0.7532 | 0.6975 |
| HumanLiver | 0.8623 | 0.8996 | **0.9202** | 0.8839 | 0.9083 |
| Adam | 0.6121 | 0.6136 | **0.7411** | 0.7125 | 0.7062 |
| Muraro | 0.8969 | 0.8999 | **0.9351** | 0.9202 | 0.7183 |
| Wang_Lung | 0 | 0 | **0.969** | 0.4287 | 0.3969 |
| Pollen | 0.9098 | 0.9169 | **0.9335** | 0.8391 | 0.8825 |
| Chen | 0.6409 | 0.6094 | **0.642** | 0.5506 | 0.5621 |
| Yan | 0.8278 | 0.7893 | **0.8783** | 0.7706 | 0.7933 |
| Camp_Liver | 0.7561 | 0.6729 | **0.7296** | 0.672 | 0.6373 |
| Camp_Brain | 0.4616 | **0.4839** | 0.4053 | 0.4128 | 0.3954 |
| Baron_Mouse | 0.6018 | **0.6556** | 0.6032 | 0.5901 | 0.4595 |
| Biase | 0.525 | 0.5887 | **0.7252** | 0.5707 | 0.5499 |
| Goolam | 0.1974 | **0.9808** | 0.9097 | 0.5826 | 0.579 |
| Baron_Human | 0.7861 | 0.7887 | **0.7906** | 0.632 | 0.6326 |
| Mouse | 0.5379 | 0.5755 | **0.5907** | 0.5304 | 0.5192 |
| Qx_Limb_Muscle | 0.8636 | **0.9284** | 0.8023 | 0.8473 | 0.7517 |
| QS_Diaphragm | 0.7395 | 0.901 | **0.9813** | 0.9699 | 0.9724 |
| QS_Heart | 0.9625 | **0.9722** | 0.6772 | 0.6078 | 0.5629 |
| QS_Limb_Muscle | 0.9144 | 0.9705 | **0.9786** | 0.9682 | 0.678 |
| QS_Lung | **0.7425** | 0.5924 | 0.6156 | 0.6142 | 0.5292 |
| Xin | 0.7103 | 0.7168 | 0.6968 | 0.8306 | **0.8398** |
| Tasic | 0.5756 | 0.5855 | 0.5689 | 0.5735 | **0.5874** |
| Average | **0.6722** | **0.7029** | **0.7518** | **0.6789** | **0.6469** |

Table S14. Comparison of the NMI values with different numbers of genes.

| Dataset | 500 | 1000 | 1500 | 2000 | 2500 | 3000 |
| --- | --- | --- | --- | --- | --- | --- |
| 10X_PBMC | 0.7238 | 0.7193 | 0.7617 | 0.7665 | 0.7661 | 0.7328 |
| Klein | 0.6098 | 0.6444 | 0.7012 | 0.7386 | 0.779 | 0.7962 |
| Human_kidney | 0.7338 | 0.7613 | 0.7488 | 0.7562 | 0.7609 | 0.7207 |
| Romanov | 0.7126 | 0.6515 | 0.6201 | 0.64 | 0.7003 | 0.6803 |
| Human1 | 0.755 | 0.8326 | 0.8771 | 0.8803 | 0.8723 | 0.87 |
| Human2 | 0.7219 | 0.8496 | 0.8714 | 0.9046 | 0.9024 | 0.8997 |
| Human3 | 0.7961 | 0.8195 | 0.8712 | 0.8189 | 0.8656 | 0.8164 |
| Human4 | 0.6493 | 0.7404 | 0.7504 | 0.7991 | 0.7633 | 0.7704 |
| Mouse1 | 0.6223 | 0.6743 | 0.6777 | 0.7833 | 0.7436 | 0.739 |
| Mouse2 | 0.6975 | 0.7583 | 0.8044 | 0.8552 | 0.8062 | 0.8001 |
| Zeisel | 0.5631 | 0.6175 | 0.68 | 0.7367 | 0.7833 | 0.8009 |
| HumanLiver | 0.8264 | 0.838 | 0.8292 | 0.8697 | 0.8338 | 0.8531 |
| Adam | 0.8018 | 0.803 | 0.7939 | 0.8038 | 0.8018 | 0.8072 |
| Muraro | 0.821 | 0.8836 | 0.8837 | 0.8961 | 0.8944 | 0.8954 |
| Wang_Lung | 0.8692 | 0.8692 | 0.8809 | 0.9233 | 0.9804 | 0.8806 |
| Pollen | 0.9406 | 0.9071 | 0.7958 | 0.9273 | 0.9084 | 0.8967 |
| Chen | 0.7424 | 0.7537 | 0.759 | 0.7655 | 0.7434 | 0.7473 |
| Yan | 0.9056 | 0.9056 | 0.9056 | 0.8966 | 0.7926 | 0.7926 |
| Camp_Liver | 0.8469 | 0.8566 | 0.8432 | 0.8492 | 0.7915 | 0.7915 |
| Camp_Brain | 0.5169 | 0.546 | 0.5434 | 0.5388 | 0.5066 | 0.5255 |
| Baron_Mouse | 0.5934 | 0.7546 | 0.7913 | 0.7969 | 0.7969 | 0.8093 |
| Biase | 0.2498 | 0.4693 | 0.5262 | 0.8267 | 0.8139 | 0.8366 |
| Goolam | 0.9184 | 0.6506 | 0.8877 | 0.8877 | 0.7248 | 0.8291 |
| Baron_Human | 0.6731 | 0.8169 | 0.8539 | 0.843 | 0.8522 | 0.8228 |
| Mouse | 0.6966 | 0.7354 | 0.7397 | 0.7596 | 0.7535 | 0.7667 |
| Qx_Limb_Muscle | 0.854 | 0.9417 | 0.8718 | 0.8731 | 0.8744 | 0.863 |
| QS_Diaphragm | 0.9194 | 0.9577 | 0.9536 | 0.9693 | 0.9693 | 0.9657 |
| QS_Heart | 0.797 | 0.8273 | 0.8824 | 0.8325 | 0.8324 | 0.838 |
| QS_Limb_Muscle | 0.9506 | 0.97 | 0.9717 | 0.9625 | 0.9564 | 0.9568 |
| QS_Lung | 0.764 | 0.7506 | 0.7721 | 0.7739 | 0.7325 | 0.7381 |
| Xin | 0.4828 | 0.5569 | 0.625 | 0.6221 | 0.6239 | 0.6246 |
| Tasic | 0.7752 | 0.7939 | 0.8067 | 0.8178 | 0.8263 | 0.8409 |
| Average | **0.7353** | **0.7705** | **0.7900** | **0.8161** | **0.8048** | **0.8034** |

Table S15. Comparison of the ARI values with different numbers of genes.

| Dataset | 500 | 1000 | 1500 | 2000 | 2500 | 3000 |
| --- | --- | --- | --- | --- | --- | --- |
| 10X_PBMC | 0.6284 | 0.6344 | 0.7604 | 0.7672 | 0.7682 | 0.6667 |
| Klein | 0.6153 | 0.6648 | 0.7206 | 0.756 | 0.7863 | 0.7965 |
| Human_kidney | 0.6862 | 0.6392 | 0.5929 | 0.6133 | 0.6168 | 0.5217 |
| Romanov | 0.7418 | 0.6472 | 0.6215 | 0.6328 | 0.7608 | 0.7362 |
| Human1 | 0.7196 | 0.8129 | 0.8435 | 0.8155 | 0.8198 | 0.8093 |
| Human2 | 0.6939 | 0.8606 | 0.8908 | 0.9129 | 0.9111 | 0.9097 |
| Human3 | 0.762 | 0.7099 | 0.6602 | 0.6389 | 0.8126 | 0.6393 |
| Human4 | 0.4543 | 0.4691 | 0.4684 | 0.6221 | 0.5078 | 0.5653 |
| Mouse1 | 0.3881 | 0.4282 | 0.4051 | 0.6209 | 0.4831 | 0.4689 |
| Mouse2 | 0.5278 | 0.5795 | 0.6039 | 0.8624 | 0.6122 | 0.6004 |
| Zeisel | 0.5053 | 0.5817 | 0.6466 | 0.7214 | 0.8133 | 0.8372 |
| HumanLiver | 0.8336 | 0.8897 | 0.8554 | 0.9202 | 0.8602 | 0.9051 |
| Adam | 0.7741 | 0.7459 | 0.7108 | 0.7411 | 0.7094 | 0.7523 |
| Muraro | 0.8717 | 0.9211 | 0.9233 | 0.9351 | 0.9322 | 0.9336 |
| Wang_Lung | 0.9392 | 0.9392 | 0.9463 | 0.969 | 0.9608 | 0.9446 |
| Pollen | 0.9507 | 0.87 | 0.6826 | 0.9335 | 0.8225 | 0.8114 |
| Chen | 0.5601 | 0.6251 | 0.5155 | 0.642 | 0.546 | 0.5602 |
| Yan | 0.8955 | 0.8955 | 0.8955 | 0.8783 | 0.6764 | 0.6764 |
| Camp_Liver | 0.7202 | 0.7382 | 0.7145 | 0.7296 | 0.6207 | 0.6207 |
| Camp_Brain | 0.3948 | 0.4165 | 0.4108 | 0.4053 | 0.3626 | 0.3861 |
| Baron_Mouse | 0.5099 | 0.5648 | 0.5983 | 0.6032 | 0.6405 | 0.6518 |
| Biase | 0.1097 | 0.2939 | 0.347 | 0.7252 | 0.6944 | 0.7491 |
| Goolam | 0.9633 | 0.4918 | 0.9097 | 0.9097 | 0.5643 | 0.6594 |
| Baron_Human | 0.5034 | 0.7692 | 0.7991 | 0.7906 | 0.8036 | 0.6525 |
| Mouse | 0.4858 | 0.5563 | 0.5348 | 0.5907 | 0.5348 | 0.594 |
| Qx_Limb_Muscle | 0.7982 | 0.9672 | 0.7979 | 0.8023 | 0.7988 | 0.761 |
| QS_Diaphragm | 0.9412 | 0.9714 | 0.9657 | 0.9813 | 0.9813 | 0.9783 |
| QS_Heart | 0.6516 | 0.6736 | 0.9038 | 0.6772 | 0.6788 | 0.6988 |
| QS_Limb_Muscle | 0.9705 | 0.9813 | 0.9846 | 0.9786 | 0.9737 | 0.9734 |
| QS_Lung | 0.6082 | 0.5145 | 0.6104 | 0.6156 | 0.4879 | 0.5091 |
| Xin | 0.593 | 0.6569 | 0.6961 | 0.6968 | 0.7235 | 0.7201 |
| Tasic | 0.4929 | 0.5133 | 0.5338 | 0.5689 | 0.5819 | 0.6221 |
| Average | **0.6653** | **0.6882** | **0.7047** | **0.7518** | **0.7139** | **0.7097** |


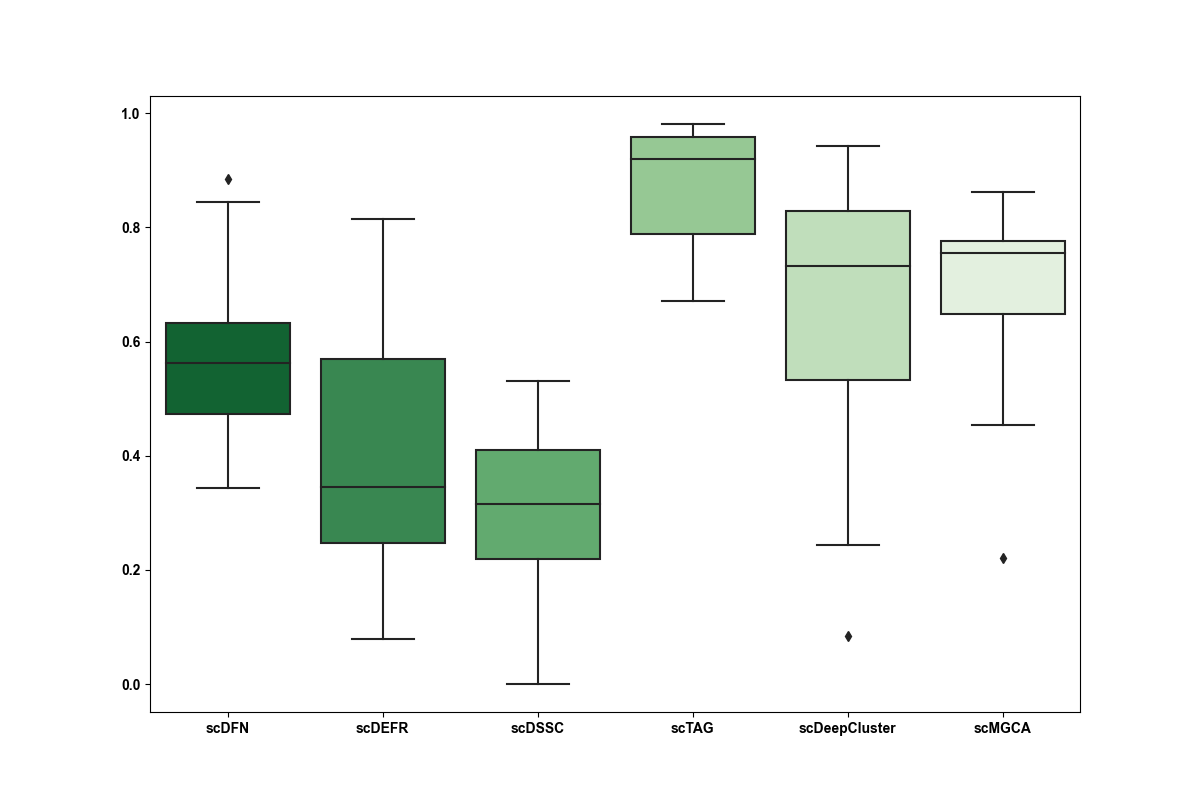


Figure S1. Clustering performance comparison of the different clustering algorithms on 32 real scRNA-seq datasets measured by ASW.


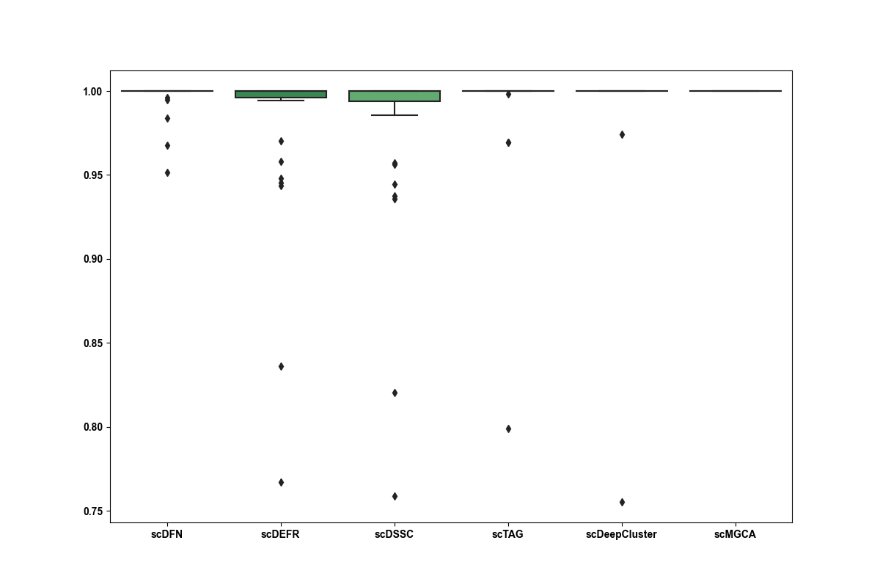


Figure S2. Clustering performance comparison of the different clustering algorithms on 32 real scRNA-seq datasets measured by cLISI.


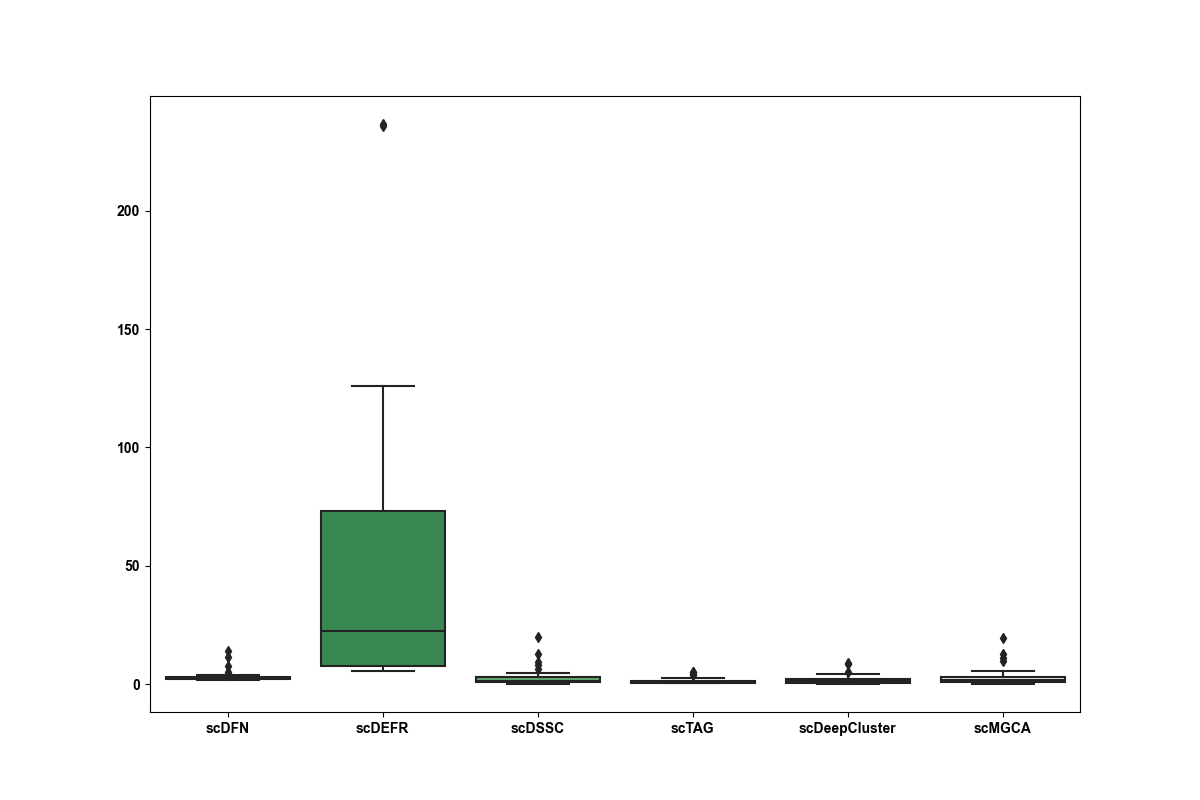


Figure S3. Running time comparison of the different clustering algorithms on 32 real scRNA-seq datasets.


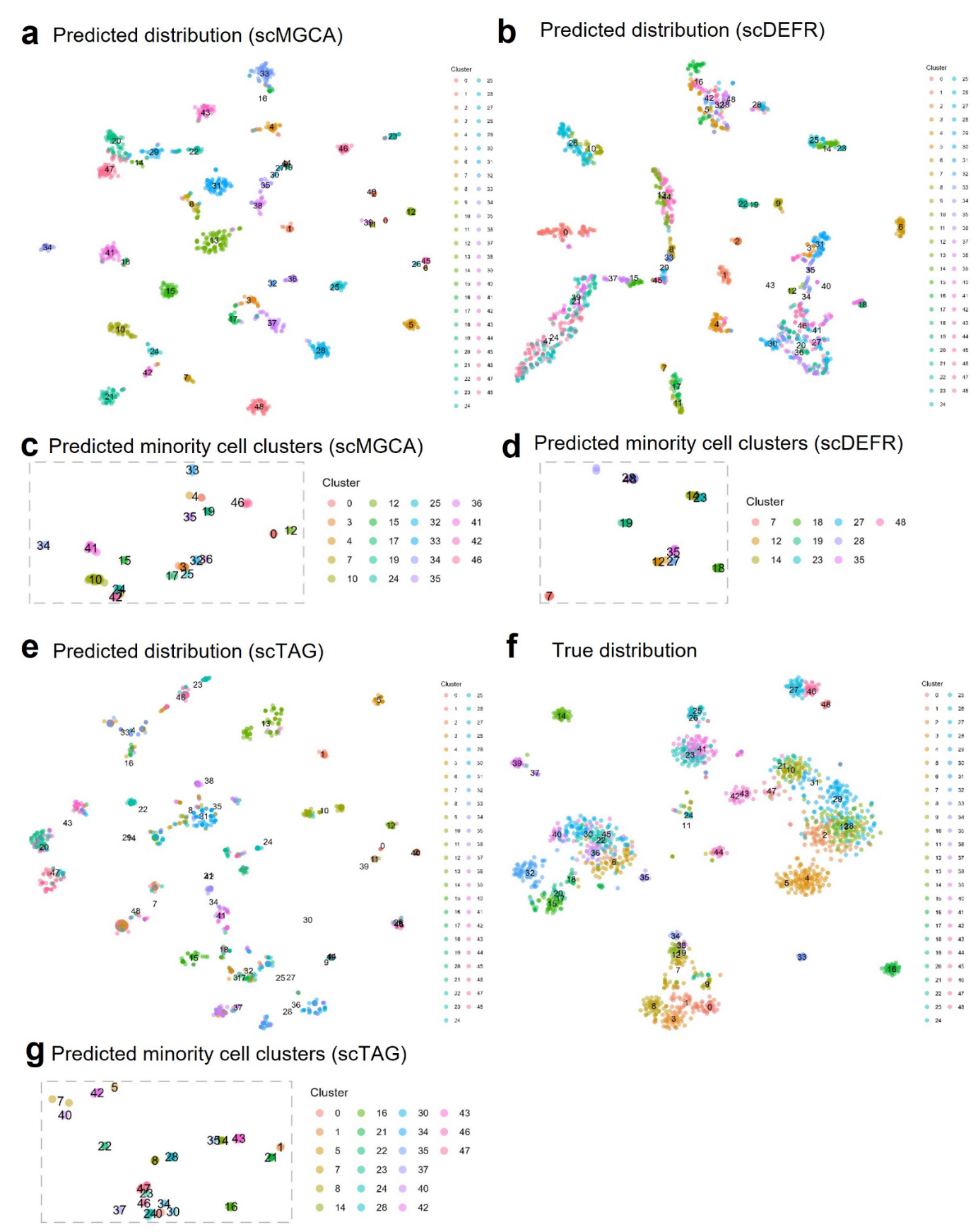


**Figure S4**. (a-g) The scDFN separated different cell types in the TASIC dataset: (a) The scMGCA prediction of 49 cell types; (b) The scDEFR prediction of 49 cell types; (c) The projection of scMGCA on small cell clusters; (d) The projection of scDEFR on small cell clusters; (e) The scTAG prediction of 49 cell types; (f) Author’s noted labels on latent space; (g) The projection of scTAG on small cell clusters.


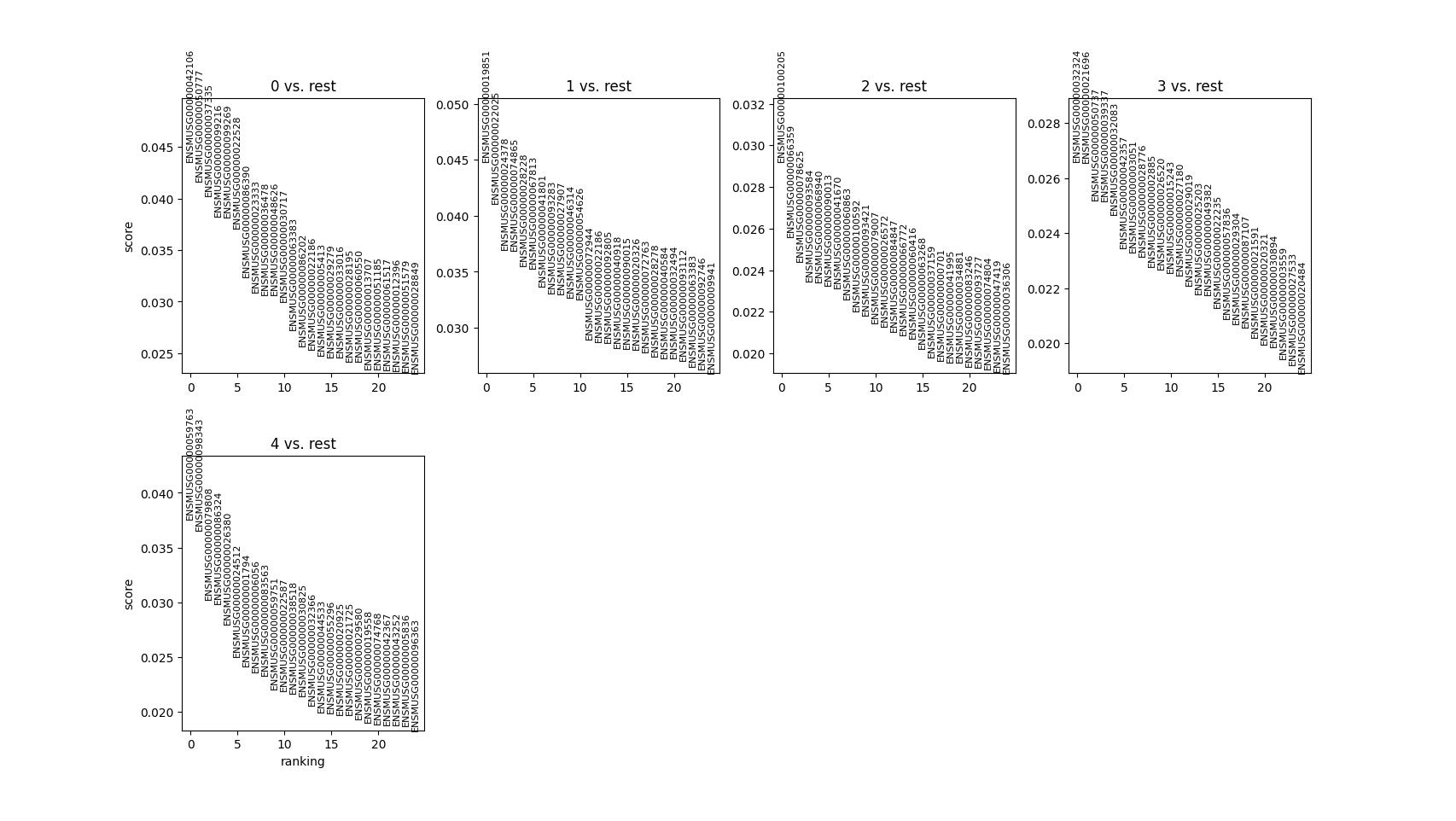


Figure S5. The results of logistic-regression-test for Goolam dataset. The five subfigures use different cell types respectively, the abscissa represents the number of genes, and the ordinate represents the test score. In each subfigure, genes are arranged from left to right by test score, showing the top 25 genes with the highest scores in each cell type.
