# Supplementary material for: scDFN: enhancing single-cell RNA-seq clustering with deep fusion networks
Source: Brief Bioinform. 2024 Oct 5;25(6):bbae486. doi: 10.1093/bib/bbae486 (PMC11456827; doi:10.1093/bib/bbae486)
Supplement: Supplementary_information_FL_bbae486 [file supplementary_information_fl_bbae486.docx]

**Supporting Information**

**1. Data collection and processing module**

We collected 32 real and public scRNA-seq datasets from multiple platforms, originating from different organs of humans and mice, such as the brain, pancreas, trachea, etc. The cell types ranged from 2 to 49, and cell numbers ranged from 56 to 12,089. These datasets were obtained from four sequencing platforms, including inDrop, 10X, SMARTer, and Smart-seq2. These datasets have known labels for each cell and have been used to test performance in previous studies. These datasets include '10X_PBMC' (SRP073767), 'Pollen' (SRP041736), 'Camp Brain' (GSE75140), 'Camp Liver' (GSE81252), 'Muralo' (GSE85241), 'Klein' (GSE65525), 'Romanov' (GSE74672), 'Adam' (GSE94333), 'Chen' (GSE87544), 'Yan' (GSE36552), 'Camp_Brain' (GSE75140), 'Camp_Liver' (GSE81252), 'Wang_Long' (GSE106960), 'Xin' (GSE81068), 'Zeisel' (GSE60361), 'Tasic' (GSE71585), 'Biase' (GSE57249), and 'Goolam' (E-MTAB-3321). The 'QS' and 'Qx' datasets are expected scRNA-seq data generated using Smart-seq2 and 10x Genomics technology in the Stanford University study. 'Baron_human' (GSE84133) is a multi-batch data set containing Human1, Human2, Human3 and Human4. 'Baron_Mouse' (GSE84133) is a multi-batch dataset. The scRNA-seq dataset supporting this study is available at <https://support.10xgenomics.com/single-cell-gene-express/datasets/2.1.0/pbmc4k>, <https://github.com/xuebaliang/scziDesk/tree/master/dataset/Young> and <https://github.com/BaderLab/scClustViz>.

The scDFN model uses the original gene expression matrix as $X\in R^{N\times d}$ input, in which *N* represents the number of cells and *d* represents the number of genes. The raw gene expression matrix was processed by the Scanpy package. We removed genes with non-zero counts in less than three cells and calculated the size factor of each cell, which is the median of the ratio of the gene expression to the geometric mean of the gene and is standardized by the cell library; thus, the total count was the same between cells. If the library size (the number of total read counts) of cell *i* is represented as *s_i_*, the cell size factor can be represented as$s_{i}/median(s)$. Since the single-cell expression matrix represents the information on gene expression levels in each cell transcriptome, all elements in the matrix have non-negative values. Therefore, we performed a logarithmic transformation to eliminate the influence of the differences in the value range of different interval data. Finally, to discard genes with low recognition and description information, the function in the Scanpy package was used to select the first *M* (*M=2000*) highly variable genes. After standardizing and compressing the data, we obtained the gene expression matrix$X'\in R^{N\times d'},d'<d$.

**2. Details regarding model implementation**

Our model was implemented using the PyTorch platform. While constructing graph neural networks, we used the KNN algorithm to construct the cell graph, and the number of nearest neighbor $k$ was set to 10, the same as that set in another study [1]. Our model training strategy was as follows. We first trained AE and IGAE independently and achieved potential embedding$\tilde{Z}$. Then, we integrated these two modules into a unified framework for pre-training to quickly obtain the optimal combination of hyperparameters. Finally, the triple self-supervised strategy was used to fine-tune the hyperparameters and obtain clustering results. Specifically, we used the classical symmetric encoder structure in AE and set the dimension reduction dimension to $M$-1024-512-128-20, the learning rate to 1e-3, and the epoch to 50 in the encoder part. We used a symmetrical graph encoder structure in IGAE and set the dimension reduction dimension to M-1024-512-128-20, the learning rate to 1e-7, the fusion parameter to 0.1, and the epoch to 50.

The module of attribute and topology information fusion consisted of pre-training and fine-training processes, whose learning rate of pre-training was 1e-5, fine-training was 1e-8, epoch of pre-training was 100, and fine-training was 50, respectively. The weight vector $\{\gamma_{1},\gamma_{2},\gamma_{3},\gamma_{4}\}$of the objective function was set to {0.3, 0.3, 0.3, 0.2}. For the parameters, the Adam optimizer was used uniformly, while learning rate attenuation was fixed. We did not set the hyperparameters for each dataset separately; instead, all test datasets used the same hyperparameters to assess the robustness of scDFN in the following sections.

**3. Autoencoder**

As learning effective cell representation is a critical step for clustering scRNA-seq data, we used an autoencoder to learn the representation of scRNA-seq data. We assumed that the autoencoder has $l$ layers, in which $l$ represents the layer number. Then, the $l$-th layer of a specific encoder process can be represented as follows:

$$H^{(l)}=\sigma(W_{e}^{(l)}H^{(l-1)}+b_{e}^{l})$$

Here, $\sigma$ represents a linear activation function, such as the Relu and Sigmoid function, $W_{e}^{(l)}$and $b_{e}^{l}$represent the weight and bias, respectively, and the initial $H^{(0)}$represents the gene expression matrix$H^{(0)}$.

The function of the decoder is to pass the data from encoding to the output layer and restore the extracted information to its original structure. The structure of encoding and decoding is symmetrical. We used the decoder to reconstruct the scRNA-seq data, which can be expressed as follows:

$$H^{(l)}=\sigma(W_{d}^{(l)}H^{(l-1)}+b_{d}^{l})$$

Here, $W_{d}^{(l)}$and $b_{d}^{l}$represent the weight and bias of the *l*-th layer. The output of the decoder is the reconstruction of the gene expression matrix, which is optimized by the MSE loss function:

$$L_{AE}=\frac{1}{2N}\sum_{i=1}^{N} \left\|x_{i}-\hat{x}_{i}\right\|_{2}^{2}=\frac{1}{2N}\sum_{i=1}^{N} \left\|X'-\hat{X}_{i}\right\|_{2}^{2}$$

**4. Performance evaluation indicators**

To evaluate the results of each clustering method, we selected two evaluation metrics for commonly used clustering. Based on the clustering results of the model, the Normalized Mutual Information (NMI) [2] and the Adjusted Rand Index (ARI) [3] can be used to evaluate the advantages and disadvantages of the model. NMI is calculated as follows:

$$NMI=\frac{I(\Omega;C)}{H(t)+H(d)}$$

$$I(\Omega;C)=\sum_{i} \sum_{j} P(\omega_{k}\cap c_{j})\log\frac{P(\omega_{k}\cap c_{j})}{P(\omega_{k})P(c_{j})}$$

Here, *H(t)* and *H(d)* represent the entropy of the true label and the detected cluster, respectively; $I$ represents mutual information and $H$ represents entropy. A more detailed description of the calculation of NMI is provided in another study [4].

The ARI is another commonly used measure to evaluate the similarity between real labels and detected clusters. Given *n* cells with *s* true clusters $C_{t}=[C_{t1},C_{t2},\cdots,C_{ts}]$ and *r* detected clusters $C_{d}=[C_{d1},C_{d2},\cdots,C_{dr}]$. The overlap between the two partitions can be summarized as a continuous table, where each entry $n_{ij}=|C_{ti}\cap C_{sj}|$represents the number of elements shared between the two clusters $C_{ti}$ and $C_{sj}$. Based on the above-mentioned information, ARI can be defined as follows:

$$ARI=\frac{\sum_{ij} \left(\begin{matrix} n_{ij} \\ 2 \end{matrix}\right)-\frac{\sum_{i} \left(\begin{matrix} a_{i} \\ 2 \end{matrix}\right)\sum_{j} \left(\begin{matrix} b_{j} \\ 2 \end{matrix}\right)}{\left(\begin{matrix} n \\ 2 \end{matrix}\right)}}{\frac{1}{2}(\sum_{i} \left(\begin{matrix} a_{i} \\ 2 \end{matrix}\right)+\sum_{j} \left(\begin{matrix} b_{j} \\ 2 \end{matrix}\right))-\frac{\sum_{i} \left(\begin{matrix} a_{i} \\ 2 \end{matrix}\right)\sum_{j} \left(\begin{matrix} b_{j} \\ 2 \end{matrix}\right)}{\left(\begin{matrix} n \\ 2 \end{matrix}\right)}}$$

Here, *a_i_* represents the sum of the row *i* of the contingency table, *b_j_* represents the sum of the row *j* of the contingency table, and ( ) represents the binomial coefficient.

**References**

1. Cheng Y, Su Y, Yu Z et al. Unsupervised Deep Embedded Fusion Representation of Single-Cell Transcriptomics, Proceedings of the AAAI Conference on Artificial Intelligence 2023;37:5036-5044.

2. Vinh NX, Epps J, Bailey J. Information theoretic measures for clusterings comparison: is a correction for chance necessary? Proceedings of the 26th Annual International Conference on Machine Learning. Montreal, Quebec, Canada: Association for Computing Machinery, 2009, 1073-1080.

3. Yu B, Chen C, Huan Y et al. scGMAI: a Gaussian mixture model for clustering single-cell RNA-Seq data based on deep autoencoder, Brief Bioinform 2021;22:1-10.

4. Hu J, Li X, Hu G et al. Iterative transfer learning with neural network for clustering and cell type classification in single-cell RNA-seq analysis, Nat Mach Intell 2020;2:607-618.
